# An ImmunoSignature test distinguishes *Trypanosoma cruzi*, hepatitis B, hepatitis C and West Nile virus seropositivity among asymptomatic blood donors

**DOI:** 10.1371/journal.pntd.0005882

**Published:** 2017-09-05

**Authors:** Michael Rowe, Jonathan Melnick, Robert Gerwien, Joseph B. Legutki, Jessica Pfeilsticker, Theodore M. Tarasow, Kathryn F. Sykes

**Affiliations:** 1 HealthTell, Inc., San Ramon, CA, United States of America; 2 HealthTell, Inc., Chandler, AZ, United States of America; Harvard School of Public Health, UNITED STATES

## Abstract

**Background:**

The complexity of the eukaryotic parasite *Trypanosoma (T*.*) cruzi* manifests in its highly dynamic genome, multi-host life cycle, progressive morphologies and immune-evasion mechanisms. Accurate determination of infection or Chagas’ disease activity and prognosis continues to challenge researchers. We hypothesized that a diagnostic platform with higher ligand complexity than previously employed may hold value.

**Methodology:**

We applied the ImmunoSignature Technology (IST) for the detection of *T*. *cruzi*-specific antibodies among healthy blood donors. IST is based on capturing the information in an individual’s antibody repertoire by exposing their peripheral blood to a library of >100,000 position-addressable, chemically-diverse peptides.

**Principal findings:**

Initially, samples from two Chagas cohorts declared positive or negative by bank testing were studied. With the first cohort, library-peptides displaying differential binding signals between *T*. *cruzi* sero-states were used to train an algorithm. A classifier was fixed and tested against the training-independent second cohort to determine assay performance. Next, samples from a mixed cohort of donors declared positive for Chagas, hepatitis B, hepatitis C or West Nile virus were assayed on the same library. Signals were used to train a single algorithm that distinguished all four disease states. As a binary test, the accuracy of predicting *T*. *cruzi* seropositivity by IST was similar, perhaps modestly reduced, relative to conventional ELISAs. However, the results indicate that information beyond determination of seropositivity may have been captured. These include the identification of cohort subclasses, the simultaneous detection and discerning of other diseases, and the discovery of putative new antigens.

**Conclusions & significance:**

The central outcome of this study established IST as a reliable approach for specific determination of *T*. *cruzi* seropositivity versus disease-free individuals or those with other diseases. Its potential contribution for monitoring and controlling Chagas lies in IST’s delivery of higher resolution immune-state readouts than obtained with currently-used technologies. Despite the complexity of the ligand presentation and large quantitative readouts, performing an IST test is simple, scalable and reproducible.

## Author summary

The most prevalent parasite infection in Latin America causes Chagas’ disease. Diagnosis is based on serologic tests because direct detection of *T*. *cruzi* is very difficult beyond its brief acute phase. The complexity of this parasite’s pathophysiology and the human immune system’s elaborate engagement with it makes current indirect diagnosis methods suboptimal. We applied the ImmunoSignature Technology to Chagas diagnosis. In addition to approximating the accuracy of current tests, a single IST test simultaneously distinguished four blood panel diseases, including Chagas. Other unique aspects include the demonstrated ability to identify subclasses within seropositive individuals. Unlike other tests, IST uses chemically-diverse peptides, not proteomic sequences, as antibody-capture ligands to identify antibody binding patterns that differ between health-states. Some of the Chagas-distinguishing ligands were enriched in sequence motifs that aligned to known *T*. *cruzi* epitopes; unmapped sequences suggest additional targets exist. While IST is also an indirect method, it holds greater capacity for delivering high-resolution serologic feedback. Relative to providing this test to the millions of individuals estimated to be infected with *T*. *cruzi*, IST is both highly scalable and reproducible.

## Introduction

Chagas’ disease ranks as the most important parasitic infection in the Western hemisphere, as measured by disability-adjusted life years lost, surpassing malaria more than 7-fold [1–5]. It is found predominantly in Latin America, but migration of infected individuals has increased its geographic distribution into Europe, North America, Japan, and Australia [5–8]. It is globally the leading cause of infectious myocarditis [8–10]. Inconsistent with this significant health burden, resources for its control are considered highly limited [11, 12]. The etiologic agent, *Trypanosoma (T*.*) cruzi*, is a flagellated protozoan transmitted predominantly via blood-feeding triatomine insects into mammalian hosts, where it can penetrate and multiply in a wide range of nucleated cell types. Other modes of dissemination include blood transfusion or congenital and oral routes [13]. Here we explore the potential application of the ImmunoSignature Technology (IST) to the diagnostic challenges of Chagas’ disease.

A week or so after infection by the trypomastigote stage protozoan, hosts experience an acute phase characterized by microscopically visible blood-parasites and tissue parasitism. Symptoms are usually mild and non-specific such that this phase is often undiagnosed; however, rare cases manifest with disease-specific periorbital swelling or ulcerative lesions at the entry site. In less than 1% of cases, the acute phase is severe and life-threatening [5]. Survivors transition into a chronically-infected phase in which the host and parasite are immunologically balanced and symptoms resolve. Most patients will remain in this clinically indeterminate stage for life, typically accompanied by the loss of detectable parasitemia, though low levels of intracellular parasites may remain measurable in certain tissues [13, 14]. From 10 to 30 years later, a third or more of these individuals will progress into a symptomatic stage of chronic disease. They suffer severe manifestations of cardiac, gastric, or other organ-related disease that lead to irreversible muscular lesions and often death [15–17]. Based on current estimates of the true prevalence of *T*. *cruzi* infection, the World Health Organization (WHO) has recently estimated that approximately 200,000 people will die from Chagasic cardiomyopathy in the next five years. A similar number of women are forecast to die in the US from breast cancer in the same timeframe [18].

The only two drugs available for Chagas treatment [7, 19] have shown limited efficacy [20, 21] and notable side-effects [19, 22]. Discovery of new drugs against *T*. *cruzi* infections that are safer and more effective [23] has been hampered by the lack of reliable, practical methods to assess efficacy in either subclinical or clinical stages of the chronic phase. There are many challenges to measuring infection status and therapeutic impact in this phase [24]. For example: i) parasitemia is subpatent and low levels of organ-concealed parasites are anatomically scattered, ii) other endemic parasites such as *Leishmania* and *Plasmodium* spp. share similar antigens with *T*. *cruzi* and iii) there are no reliable biomarkers of incipient or active disease [25]. In summary, there is need for a test that stratifies *T*. *cruzi* seropositive individuals into clinically distinct groups. These strata include distinguishing individuals who remain infected from those that manage to resolve a chronic phase infection. It would also be desirable to predict which indeterminate-stage individuals will remain asymptomatic from those that will progress to suffering life-threatening complications. A recent study identified four biomarkers that added predictive value of short-term mortality in patients suffering severe cardiomyopathy [26]. Another study identified a set of inflammatory cytokines and cardiac biomarkers that were elevated in patients with severe disease [25]. A transcriptome analysis identified a set of genes differentially expressed between patients with moderate versus severe Chagas cardiomyopathy [27]. Together, these results suggest that molecular stratification of clinically distinct patient groups is possible.

The WHO recommends diagnosis of disease to be based on epidemiological risk and laboratory testing [28]. In the acute phase, direct detection of parasite is possible; however, this is not reliably sensitive in the chronic phase. For these individuals, diagnosis is based on indirect positive detection by two serologic tests [29]. In 2007, US blood centers began serologically screening blood and organ donors. The FDA has approved the Ortho *T*. *cruzi* ELISA [30] and Abbott PRISM [31] tests. These report a signal to cut off value (S/CO) that quantifies levels of lysate-antigen binding in blood plasma and reflects antibody titers. A confirmatory radio-immunoprecipitation assay (Quest Diagnostic *T*. *cruzi* RIPA) [32] is employed routinely by many blood banks [30].

Newer generation assays are under development based on various mixtures of recombinant proteins. In a study to screen for new antigen candidates, a tiled peptide array spanning 457 *T*. *cruzi* proteins was probed against pooled immunoglobulin G (IgG) preparations purified from *T*. *cruzi-*antibody positive sera. Binding analysis identified 97 novel candidate-antigens [33]. Since this eukaryotic pathogen has a complex proteome and life cycle [34], many targets may be needed to fully capture human immune responses to it [8]. A pre-requisite for developing a test with new Chagas’ disease-related diagnostic capacities would seem to be access to a reliable and scalable platform for measuring the diversity of host responses to *T*. *cruzi* infections.

The ImmunoSignature Technology has shown applicability to the classification of many immune-mediated diseases [35–40]. It is based on diverse yet reproducible patterns of peripheral antibody binding to an array of >100,000 peptides that were selected to provide an unbiased sampling of all possible amino acid (a.a.) combinations rather than to correspond to any biological sequences. The assay is performed with a small sample of blood, plasma, or serum [41]. A peptide bound by an antibody is presumably not the original antibody recognition sequence but rather mimics the sequence or structure of the epitope. Since the diversity of chemical sequences is several orders of magnitude greater than proteomic sequences, a broad range of mimicry is afforded. For example, a chemical-library peptide may be mimicking a linear sequence, a structure, a mutated sequence such as found in tumors, or a non-peptidic biomolecule such as carbohydrate. Even if the cognate epitope is peptidic and linear, the probability of any mimetic-peptide (mimotope) exactly matching the epitope is low because the arrayed library of peptides only samples chemical sequence space. Each IST peptide sequence that is selectively bound by an antibody is a functional surrogate of an epitope that the antibody recognizes *in vivo*. When the mimotope signal is unique to a health state, the bound antibody becomes a biomarker. Collectively, all specifically-bound antibodies correlated with a given health state are informative for both detecting and monitoring it. A key advantage of this platform is that it is not designed to accommodate any single disease, such that the same library is universally appropriate. In principle, it should be informative for any condition that elicits a specific immune response once a unique, multivariate algorithm is generated. Application-specific accommodations can be reserved as optimization steps, if desired. In demonstration of this universality, we conducted two studies with the same combinatorial peptide library. In the first, a binary disease positive versus negative contrast was evaluated. In the second, the ability to simultaneously distinguish between multiple infectious diseases with one algorithm was explored. For this work, we chose to evaluate the ability of IST to classify individuals who were seropositive but asymptomatic for *T*. *cruzi*, hepatitis B virus (HBV), hepatitis C virus (HCV) and West Nile virus (WNV) or seronegative for all four. Blood specimens from these donors were available that had been retrospectively collected using the same protocols, and declared positive or negative by the same testing regimens.

The main goal of this work was to establish the feasibility of using IST to distinguish serologic *T*. *cruzi* positivity in asymptomatic blood donors that either were *T*. *cruzi* seronegative or were seropositive for a different disease. The studies presented here demonstrate IST as a viable diagnostic approach for *T*. *cruzi* and lay the groundwork for its use in new areas of clinically relevant discovery.

## Methods

### Donor samples

Plasma samples were collected by United Blood Services (http://www.unitedbloodservices.org), and obtained from Creative Testing Solutions (CTS, Tempe, AZ) in 2015 as samples that were blood panel tested as being serologically-positive for *T*. *cruzi-*specific antibodies and corresponding age and gender matched samples that were determined to be *T*. *cruzi* antibody-negative. A second cohort of *T*. *cruzi* seropositive and seronegative samples were obtained from CTS in 2016. These specimens tested negative for all other blood panel diseases. Additional plasma samples that serologically tested positive for HBV, HCV and WNV were obtained from CTS in 2016. These were all collected by United Blood Services in the US, from the blood donor population; ethnicity is provided for those for which it was reported to CTS. Upon receipt the specimens were thawed, a portion of each was removed and mixed 1:1 with ethylene glycol as a cryoprotectant, then aliquoted into single-use volumes. Aliquots were stored at -20°C until needed. The remaining undiluted sample volume was stored neat at -80°C. Identities of all samples were tracked using 2D barcoded tubes (Micronic, Leystad, the Netherlands). In preparation for the assay, sample aliquots were warmed on ice to 4°C and diluted 1:100 in primary incubation buffer (Phosphate Buffered Saline with 0.05% Tween 20 (PBST) and 1% mannitol). Microtiter plates containing the 1:100 dilutions were then diluted to 1:625 for use in the assay. For the subset of samples selected for evaluating platform performance across wafer lots, the 1:100 dilutions were aliquoted into single-use microtiter plates and stored at -80°C. All aliquoting and dilution steps were performed using a BRAVO robotic pipetting station (Agilent, Santa Clara, CA). All procedures, which used de-identified, banked plasma samples, were reviewed by the Western Institutional Review Board (protocol no. 20152816).

### Arrays

A combinatorial library of 125,509 peptides with a median length of 9 residues and range from 5 to 13 a.a.’s was designed to include 99.9% of all possible 4-mers and 48.3% of all possible 5-mers of 16 a.a.’s. Methionine and cysteine were excluded because of their oxidation and cyclization potential, which would cause probe variability. Isoleucine and threonine were excluded because valine and serine, respectively, are generally considered chemically and structurally similar. Valine and serine were selected for inclusion because they are structurally smaller than their excluded counterpart. Limiting the number of amino acids reduced fabrication complexity and cost while maintaining a survey of a similar diversity of chemical and structural space. Several categories of control peptides added 6,203 features. For example, 500 features corresponded to the established epitopes of five different well-characterized murine monoclonal antibodies (mAb), each replicated 100 times. Another 935 features corresponded to four different sequence variants of three of the five established epitopes, each replicated from 100 to 280 times. Another 500 control peptides were designed with a.a. compositions like those of the library peptides, but were uniformly 8-mers in length and present in triplicate. The median signals of these 1500 8-mers were quantitated and treated as part the library when developing the IST models; the other control peptides were not. The binding of purified murine antibodies, antibody-spiked human plasma, and cohorts of normal donor plasma samples to all of these control features were analyzed in platform validation studies. They were also monitored in all library-directed assays conducted with IST study samples to ensure data quality. The remaining 3,268 controls include fiducial markers to aid grid alignment, analytic control sequences and linker-only features. Except for the fiducials, all features are distributed evenly across the array. The distribution of replicate features enabled spatial variability to be analyzed by measuring the coefficient of variance of peptides with many or few replicates; it also enabled the detection of any signal gradients or other aberrancies. Finally, the library peptides themselves served as a category of control features. Namely, on and off target specificity assessments were made by analyzing the binding of mAbs with known epitope recognition to the diverse panel of library peptides.

The peptides were synthesized on a 200 mm silicon oxide wafer using standard semiconductor photolithography tools adapted for *tert*-butyloxycarbonyl (BOC) protecting group peptide chemistry, using methods described previously [40]. Briefly, an aminosilane functionalized wafer was coated with BOC-glycine. Next, photoresist containing a photoacid generator, which is activated by UV light, was applied to the wafer by spin coating. Exposure of the wafer to UV light (365nm) through a photomask allows for the fixed selection of features on the wafer to be exposed with any given mask. After exposure to UV light, the wafer was heated, allowing for BOC-deprotection of the exposed features. Subsequent washing, followed by the application of an activated a.a. would complete a cycle. Each cycle added a specific a.a to the N-terminus of peptides located at masked-defined locations within the array. These cycles were repeated, varying the mask and a.a.’s that were coupled at each feature, to obtain the combinatorial library of chemical sequence peptides. The arrays used in the Chagas study were synthesized on a hydrophilic wafer surface, (3-glycidoxypropyl)trimethoxysilane-2,2’-(ethylenedioxy)bis(ethylamine), with a polyethylene glycol linker. The arrays in the multi-disease study were synthesized on a less hydrophillic wafer surface, (3-glycidoxypropyl)trimethoxysilane -poly(allylamine), with a SGSG linker.

Each completed wafer was diced into 13 rectangular regions each having the dimensions of standard microscope slides (25mm x 75mm). Each of the slides contained 24 arrays arranged in eight rows by three columns. Finally, the protecting groups on several a.a. side chains were removed using a standard cocktail [42]. The fully prepared slides were stored in a dry nitrogen environment until used in assays.

Several quality tests were performed to ensure arrays were manufactured within process specifications including the use of 3σ statistical limits for each step. Wafer batches were sampled intermittently by MALDI-MS to verify that each a.a. was being coupled at the intended step with efficiencies of >97% (95%-100%), thus ensuring that the final combinatorial synthesis products were correct. Wafer manufacturing was tracked from beginning to end via an electronic custom Relational Database. Data typically tracked include chemicals, recipes, time and technician performing tasks. After a wafer was produced the data was reviewed and the records were locked and stored. Finally, each lot was evaluated in a binding assay to confirm performance, as described below.

Our current automated system, using commercially available wafer-synthesis tools, is capable of manufacturing 1,248 arrays in three days. Production could be scaled by adding more shifts or purchasing additional automated systems.

### Monoclonal assay

Prior to conducting the IST assays with donor plasma, the binding activity of commercial, murine monoclonal antibodies (mAb) was evaluated against control peptides corresponding to the established recognition sequence of each mAb. The IST arrays were probed separately, in triplicate, with 2.0 nM of antibody clones 4C1 (GeneTex, Inc., Irvine, CA), p53Ab1 (EMD Millipore, Billerica, MA), p53Ab8 (EMD Millipore) and LnkB2 (Absolute Antibody, Ltd., Cleveland, United Kingdom) in primary incubation buffer (1% mannitol, PBST), as detailed below. Binding was detected with 4.0 nM goat anti-mouse IgG conjugated to DyLight 549 (KPL, Inc., Gaithersburg, MD) and signal was quantified, as detailed below.

### Plasma assay

Production quality manufactured microarrays were rehydrated prior to use by soaking with gentle agitation in distilled water for 1 h, PBS for 30 min and primary incubation buffer (1% mannitol, PBST) for 1 h. The microarray slides were next briefly rinsed in distilled water to prevent a surface salt residue and centrifuged to remove excess liquid. The slides were loaded into an ArrayIt microarray cassette (ArrayIt Corporation, Sunnyvale, CA) to adapt the individual slides into a microtiter plate footprint. Using a liquid handler, 90μl of each sample was prepared as a 1:625 dilution in primary incubation buffer (1% mannitol, PBST) and then transferred to the cassette. This mixture was incubated on the arrays for 1 h at 37°C with mixing on a TeleShake95 (INHECO, Martinsried, Germany) to drive antibody-peptide binding. Following incubation, the cassette was washed three times in PBST using a BioTek 405TS Select microtiter plate washer (BioTek Instruments, Inc., Winooski, VT). Bound antibody was detected using either 4.0 nM goat anti-human IgG (H+L) conjugated to AlexaFluor 555 (Invitrogen-Thermo Fisher Scientific, Inc., Carlsbad, CA), or 4.0 nM goat anti-human IgA conjugated to DyLight 550 (Novus Biologicals, Littleton, CO) in secondary incubation buffer (0.5% casein in PBST) for 1 h with mixing on a TeleShake95 platform mixer, at 37°C. Following incubation with the secondary antibody, the slides were again washed with PBST, followed by distilled water; once removed from the cassette, the slides were sprayed with isopropanol and centrifuged dry. Quantitative signal measurements were obtained by determining a relative fluorescent value for each addressable peptide feature. These assay conditions were identified in several design of experiment (DOE) matrices in which plasma dilution, secondary concentration, blocking reagent, incubation times, and shaking fluidics were permutated.

Separately, ELISAs were conducted to assess cross-reactivity between the anti-IgG and anti-IgA secondary antibody products used in this work. A low level of cross-reactivity was noted for the anti-IgG product against the IgA secondary; no reactivity was detectable by the anti-IgA product against the IgG secondary reagent.

### Data acquisition

Assayed microarrays were imaged using an Innopsys 910AL microarray scanner fitted with a 532nm laser and 572nm BP 34 filter (Innopsys, Carbonne, France). The Mapix software application (version 7.2.1; Innopsys) identified regions of the images associated with each peptide feature using an automated gridding algorithm. Fluorescent intensities were acquired from 14 laser line-scans at 1μm resolution for each feature of the array. Median pixel intensities for each peptide-feature were calculated and saved as a tab-delimitated text file, and stored in a database for analysis.

### Binding data analysis

The median feature intensities were log_10_ transformed after adding a constant value of 100 to improve homoscedasticity. The log-transform was applied because the data were log-normally distributed; the precision of the measurements was approximately proportional to the intensities. The intensities on each array were normalized by subtracting the median intensity of the combinatorial library features for that array.

For analysis of the monoclonal assays, selective binding of each mAb to its cognate epitope was assessed using a Z-score, calculated as:

$$\text{Z}=\frac{mean({\text{I}}_{mAb})-mean({\text{I}}_{2{}^{\circ}})}{sd({\text{I}}_{2{}^{\circ}})}$$

where I_mAb_ and I_2o_ are the transformed peptide intensities in the presence of mAb and secondary or secondary reagent only, respectively. Binding intensities of all four mAbs to all features corresponding to the four epitope sequences were measured. Calculated scores were based on scanner measurements with a range from 0 to 65,535 RFI units.

For analysis of the IST assays, binding of plasma-antibodies to each library feature, as detected by fluorescently labeled secondary antibodies, was measured for all donor samples. A complete list of all library peptides and the raw fluorescent intensities measured at each library peptide-feature for each of the donor samples used in classifier development are reported in the Dryad Digital Repository (http://dx.doi.org/10.5061/dryad.p6882). From these data, peptides were identified as displaying differential signal levels between different health groups by using a t-test of mean peptide intensities of a peptide within a group to that of the contrasting group. The Welch adjustment for unequal variances was applied. For the 2015 Chagas cohort, *T*. *cruzi* seropositive donors (n = 146) were compared to seronegative donors (n = 189), and peptides with significantly differential signal were identified. In a separate experiment, a set of peptides was identified that co-discriminated Chagas and three viral-positive donor samples. The mean intensities were compared of Chagas positive (n = 88), HBV positive (n = 88), HCV positive (n = 71) and WNV positive (n = 88) donor samples, as determined by blood panel testing protocols. For both the Chagas and the multi-disease experiments, peptides that showed significant discrimination were identified based on 5% threshold for false positives after applying the Bonferroni correction for multiplicity (i.e., *p* <4e-7). Even though peptide selection is done at the peptide level, our classification analysis does not depend on the performance of individual peptides, but rather on the power of the multivariate peptide classifier.

In another analysis of the 2015 cohort results, a Pearson correlation was calculated for the transformed peptide intensities of Chagas-positive donors to their median signal over cut-off value (S/CO) from three serially conducted *T*. *cruzi* ELISA assays. Library peptides correlated to S/CO were identified using a 10% false discovery rate (FDR) criterion by the Benjamini-Hochberg method [43] or by the Bonferroni correction for multiplicity.

To construct the Chagas and multi-disease experiment classifiers, features were ranked for their ability to discriminate samples based on the *p* value associated with a Welch’s t-test that compared Chagas positive to negative donors or that compared all four different disease classes, respectively. The number of peptides selected was varied from 5 to 4000 features progressively. The transformed intensities of each peptide were mean centered and scaled to unit variance. To train a classifier, these data were input to a support vector machine (SVM) [44] with a linear kernel and cost parameter of 0.01. A four-fold or five-fold cross validation procedure was repeated 100 times and used to assess model performance. This was estimated as the area under the receiver-operating characteristic (ROC) curve, which incorporated both feature selection and classifier training steps to avoid bias.

A classifier was fixed that comprised an optimal number of ranked input peptides based on the cross-validated performance estimates of the 2015 Chagas cohort. This fixed SVM classifier model was evaluated without further optimization (fixed coefficients) by using it to predict the classification of samples in the independent 2016 Chagas cohort, which served as a test set for verification of the algorithm. The blood bank’s positivity assignments of these test samples were blinded to the researchers conducting the IST assay until the results were obtained. This classifier was also used in assessing signal precision and performance reproducibility of the platform.

All analyses were performed using R version 3.2.5 [45]. SVM classifiers were developed using the e1071 package [46].

### Peptide alignment scoring

Library peptides were aligned to the *T*. *cruzi* CL Brener [47] and Sylvio proteomes downloaded October 2016 from UniProt. A BLAST strategy [48], requiring a seed of 3 a.a.’s, a gap penalty of 4 a.a.’s, and a scoring matrix of BLOSUM62 [49] modified to reflect the a.a. composition of the array [50]. These modifications increased the score of conserved substitutions, removed penalties for a.a.’s absent from the array and scored all exact matches equally.

An alignment score is assigned to each a.a. position of a protein sequence to which a given set of peptides align; the overlap score is the sum of these a.a. alignment scores. To correct this score for library composition, another overlap score was calculated using the identical method but for a list of all array peptides. This allowed for the calculation of a peptide overlap difference score, **s**, at each a.a. position via the equation:

s=a−(b/d)*c

In this equation, **a** is the overlap score from the classifying peptides, **b** is the number of classifying peptides, **c** is the overlap score for the full library of peptides and **d** is the number of peptides in the library.

To convert these s scores (which were at the a.a. level) to a full-protein statistic, the sum of these scores for every possible 20-mer epitope tiled acrossa protein was calculated. The final protein epitope score, S, was the maximum score along the rolling window of 20-mers for each protein. A similar set of scores was calculated for 100 iterative-rounds of randomly selected peptides from the library, equal in number to the number of classifying peptides. The *p*-value for each score S was calculated by permutation test from the number of times this score was met or exceeded among the randomly selected peptides, controlling for the number of iterations.

### Precision analyses

The precision of antibody binding to the array features was characterized using a set of eight plasma samples to measure the fluorescent signals of the library peptides that comprised the fixed model for classifying *T*. *cruzi* seropositive (Chagas positive) versus seronegative (Chagas negative) donor samples. The fixed classifier was fit using data from the 2015 cohort and then applied to these slides without any further optimization. Four Chagas positive samples associated with a range of median ELISA S/CO values and three Chagas negative samples were selected from the 2015 cohort of donors, and assayed in triplicate. A well-characterized in-house plasma sample from a healthy donor was also included in the slide design, assayed in duplicate. As a negative control, one array was assayed in the absence of plasma during the primary incubation step but in the presence of the secondary detection antibody. These 24 samples were assigned to array positions on a single slide such that replicates were evenly distributed. This slide layout was repeated across slides according to the designs specified below. Signal precision of the peptides comprising the fixed classifier was determined for each study. The normalized readouts were fit to a mixed effects model from which inter-array, inter-slide, inter-wafer, inter-day, and inter-wafer batch CVs were calculated. Each donor sample was treated as a fixed effect. The nested factors ‘wafer’ or ‘wafer batch’, ‘slide’, and ‘array’ were crossed with ‘day’, and these were treated as random effects. Models were fit in R using the lme4 package [51] to derive coefficients of variance (CV).

To evaluate precision within a manufacturing batch, three wafers from a single batch were selected. Twelve of the thirteen slides from each of these wafers were evaluated using the one-slide precision design described above. The 36 slides were evaluated across three ArrayIt cassettes, which each carry four-slides, on three different days. Slides from each wafer were assigned evenly across the three days such that each cassette contained two slides from one of the three wafers and one slide each from the remaining two wafers.

To measure precision across wafer batches, one wafer was selected from each of four different manufacturing batches. Twelve of the thirteen slides from each wafer were evaluated using the precision study sample-set described above. These slides were distributed for testing across four cassettes per day, spanning three days. Slides from each wafer were distributed evenly across the three days such that each cassette contained two slides from two of the four wafers.

### Performance analyses

As an internal QC assessment of the robustness of the fixed Chagas classifier across many wafer manufacturing batches and assay days, a quality control (QC) sample panel was designed for testing on a single slide. The layout comprised a representative panel of 11 Chagas positive samples, 11 Chagas negative samples, a well characterized in-house healthy donor sample and a secondary-only array. This panel was assayed on a slide from 22 different wafers manufactured across 10 different synthesis batches. For each of the 22 wafer-slides tested, the fixed model classifier developed in the Chagas 2015 trial was applied to the QC set of 22 Chagas positive or negative samples to estimate area under the ROC curve (AUC). One of these wafers included in this analysis was used for the Chagas study (Chagas positive and negative) and another was used for the multi-disease study (*T*. *cruzi*, HBV, HCV, & WNV) trial.

## Results

### Platform validation

A peptide synthesis protocol has been developed in which many a.a. coupling reactions are performed in parallel directly on silicon wafers using masks and photolithographic techniques. In the work presented here, arrays displaying a total of 131,712 peptides (median length of 9 a.a.) *in situ* synthesized at features of 14 μm x 14 μm were utilized to query antibody-binding events. The array layout includes 125,509 library-peptide features and 6203 control-peptide features attached to the surface via a common linker (see Methods). The library peptides were designed to provide a sampling of all possible a.a. combinations and were not designed to match any proteomic sequences. Several sets of control-features were included in the array. These controls provided synthesis verification of intended peptide sequences, precision and reproducibility measurements, and fiducials for manufacturing and data processing.

**Fig 1:**
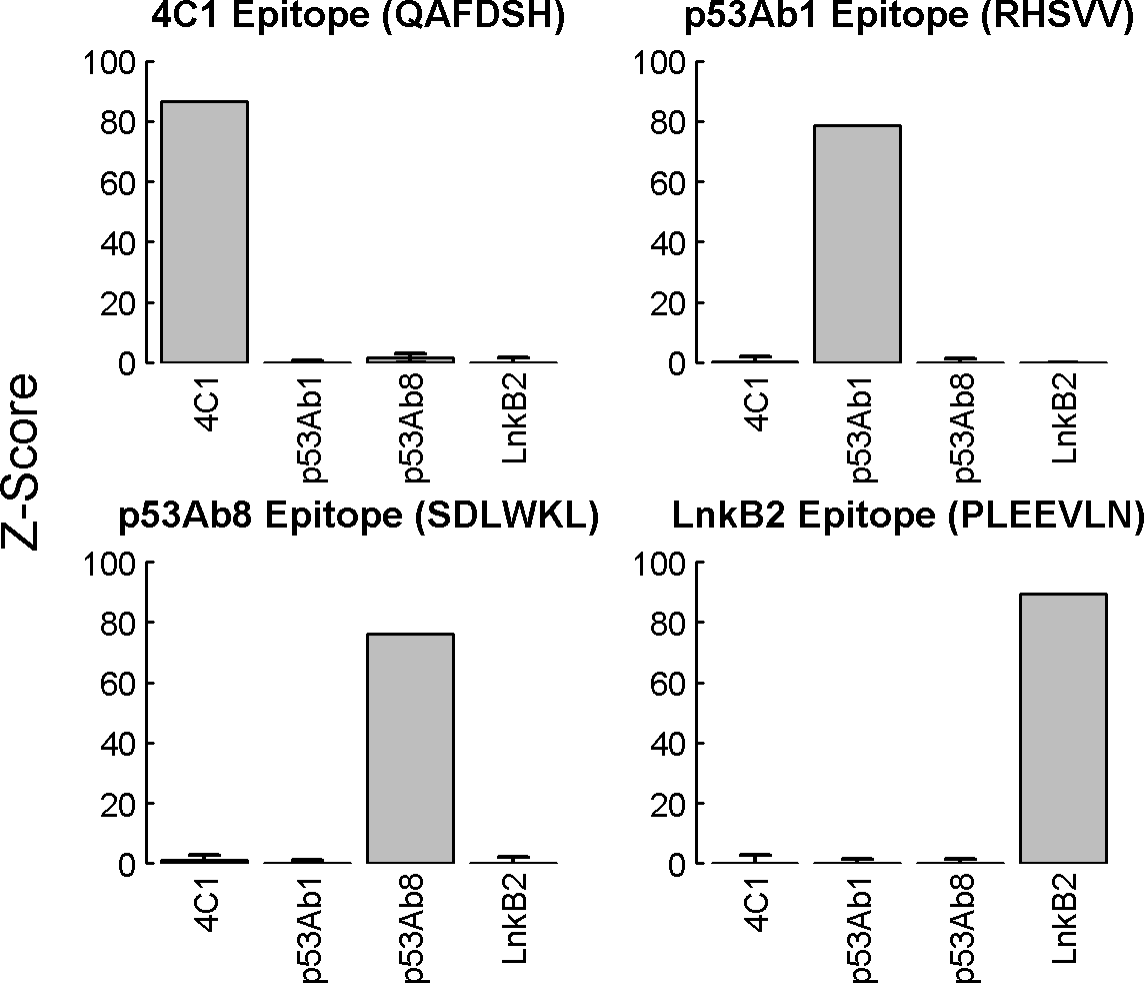
Binding of monoclonal antibody standards to cognate-epitope control features. Four well-characterized mAbs (4C1, p53Ab1, p53Ab8 and LnkB2) were separately applied to arrays at 2.0 nM with competitor, in triplicate, and then detected with an anti-mouse secondary reagent. For each binding assay the mean log_10_ relative fluorescent intensity (RFI) of the epitope control features were used to calculate a Z-score. The results corresponding to each epitope were plotted as separate bar graphs, with the mAb recognition sequences shown above the graphs. The mAb clones are indicated as bars along the x axes within each graph. Error bars represent the standard deviation of the individual control feature Z-scores. Cognate controls were saturated, leaving a standard deviation of zero on the Z scores for these feature replicates.

Experiments were conducted using mAbs that functionally evaluated the quality of final array-synthesized products with respect to ligand presentation and antibody recognition. A panel of four murine antibody clones (4C1, p53Ab1, p53Ab8, and LnkB2) were selected with recognition sequences that correspond to four of the five epitope control-peptides designed within the array layout. These four epitopes collectively include all 16 a.a. that were used to synthesize the library. Fig 1 presents the results from a binding assay conducted as described (see Methods) in which each antibody was individually applied to an array with competitor agent, in triplicate. For each mAb, the control feature intensities were used to calculate a Z score for both the peptide sequence corresponding to its epitope, and the three non-cognate sequences. Each of the cognate sequences were bound with high signal intensity whereas the non-cognates displayed little or no signal above background values (secondary antibody only). The dynamic ranges (95^th^ percentile/5^th^ percentile) of the raw intensities of the library features were ~1.04, reflecting binding to less than 1% of the library peptides. This indicates that the microarrays carry peptides suitable for specific antibody recognition and binding.

### Chagas cohorts

Two cohorts of plasma samples collected from asymptomatic donors were obtained from a blood bank repository (Creative Testing Solutions (CTS), Tempe, AZ), and are described in S1 Table. CTS tests all blood collected by United Blood Services (UBS) against a panel of infectious diseases. The 2015 cohort is comprised of 335 donors that were each serologically tested for *T*. *cruzi*-specific antibodies using the blood bank’s algorithm. Three ELISAs were serially performed to assay plasma against *T*. *cruzi* whole cell lysate (Ortho ELISA Test System). If any one of these scored positive by a signal to cutoff value (S/CO > 1.0), then a confirmatory test was performed. The confirmatory test was an immunoprecipitation assay (Quest Diagnostic RIPA test) that uses plasma to precipitate radiolabeled *T*. *cruzi* lysates. By these criteria 146 donors were determined to be T. *cruzi* seropositive, and were declared “Chagas positive” by the blood bank; 189 were determined to be *T*. *cruzi* seronegative, and were declared “Chagas negative”. This designation is widely used, although they are subclinical and it is understood that these Chagas positive individuals may or may not have remained parasite-infected at the time of the draw. A more accurate designation would be *T*. *cruzi*-antibody positive or negative. Literature sources consider an S/CO score above 4.0 to be strong positivity [52]; this assigns 49 of the 146 (33.5%) seropositive donors into a strongly positive S/CO subgroup and 66.5% as weakly positive. The distributions of gender, age, and ethnicity were those typically observed in the UBS blood donor population (http://www.unitedbloodservices.org/aboutUs.aspx), although not all demographic information was reported. The 2016 cohort comprised 116 donors that were tested for *T*. *cruzi-*antibodies with the same protocol of ELISA and RIPA testing described above. This second cohort was designed to contain 58 Chagas positive and 58 Chagas negative participants. A somewhat higher proportion of the seropositive individuals scored into the higher S/CO subgroup, 31 of 58 (53%). The distributions of gender, age, and ethnicity were like those of the 2015 cohort, with ethnicity reporting being more complete (89% versus 62%). The blood donors described here were located predominantly in the US West, reflective of the bias for Hispanic over black minority participants.

The study trial presented here was conducted by using the 2015 cohort as an algorithm-training set to be used for developing a classifier that distinguishes *T*. *cruzi* seropositive from seronegative individuals. This classifier was fixed and then applied to the prediction of positivity for the 2016 cohort donor samples. Thus, analysis of the 2016 cohort served as a training-independent verification trial.

### Performance of IST in determining Chagas positivity

**Fig 2:**
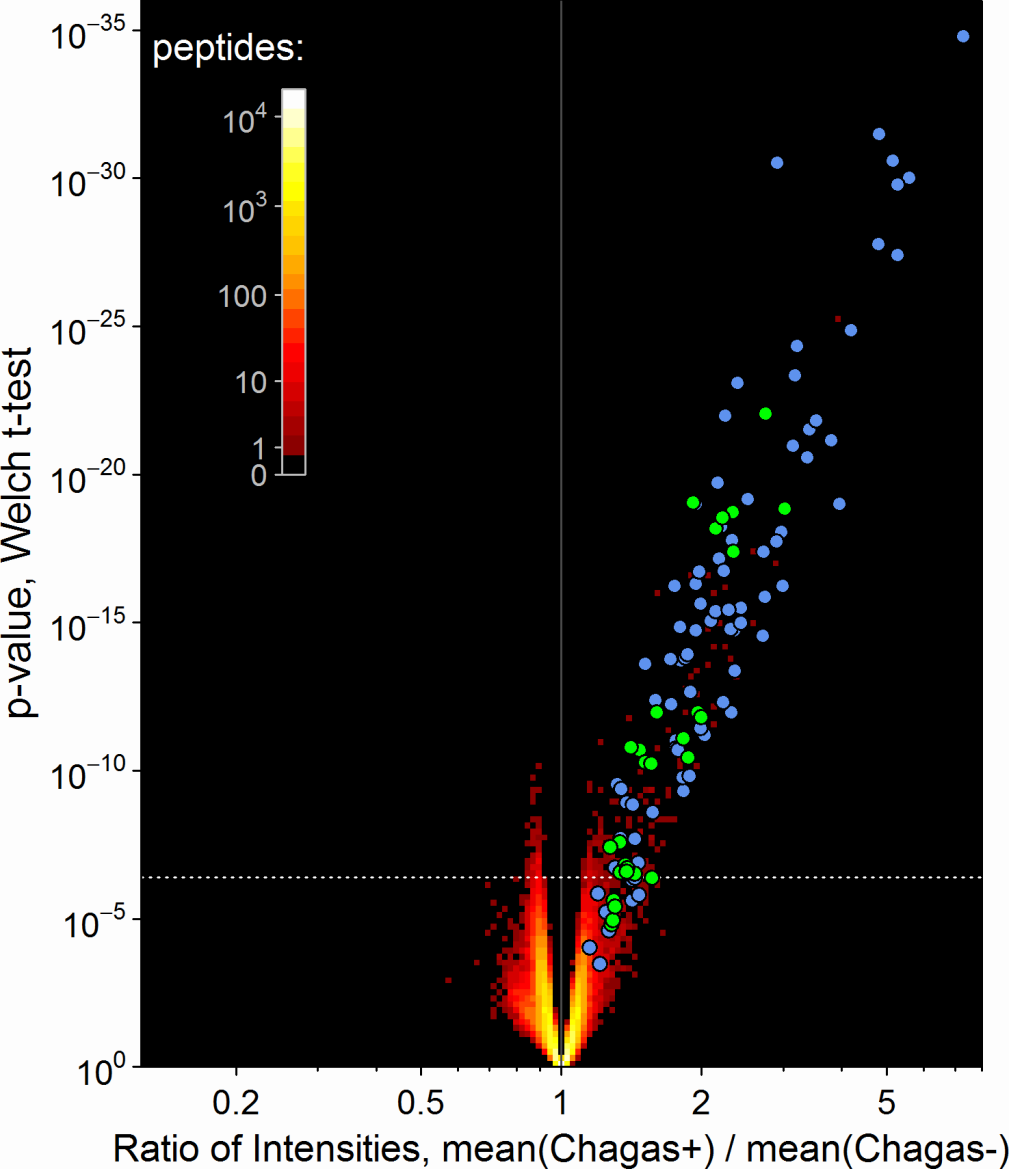
A set of library peptides reproducibly displayed antibody-binding signals that were significantly different between Chagas seropositive and seronegative donors. A volcano plot is used to assess this discrimination as the joint distribution of t-test *p*-values versus the ratio of geometric mean intensities for Chagas positive donors relative to Chagas negative. The density of peptides at each plotted position is indicated by the color scale. The 356 peptides above the dashed white line discriminate between positive and negative Chagas sero-status by IST with 95% confidence after applying a Bonferroni adjustment for multiplicity. The colored circles indicate individual peptides with intensities that were significantly correlated to the *T*. *cruzi* ELISA-derived S/CO value either by a Bonferroni threshold of *p* < 4e-7 (green) or the less stringent false discovery rate (FDR) of <10% (blue).

All IST assays were performed as described (Methods) and scanned to acquire signal intensity measurements at each feature. Application of Welch’s t-test identified 356 peptide sequences that had significant differences in mean signal between those donors who were blood-bank scored as seropositive versus seronegative for *T*. *cruzi-* specific antibodies. The volcano graph in Fig 2 plots the ratio of mean signal intensities between seropositive and negative samples versus the significance of the differential, for each peptide on the array. A white dashed line demarcates the Bonferroni-corrected *p* value limit. Most, though not all, of the significantly different peptide signals displayed higher binding intensities in the seropositive as compared to seronegative donor samples. Approximately half of these class-distinguishing peptides had signal levels that were also positively correlated to the median *T*. *cruzi* S/CO value of donor samples declared Chagas positive (shown as blue and green circles). This is consistent with the possibility that some library peptides bind the same or related plasma-antibodies as those bound by antigen in the ELISA screen. Conversely, there were 14 peptides that were significantly correlated to the S/CO value but did not meet the Bonferroni threshold for IST discrimination of Chagas positivity (circles below white dashed line). The remaining half of the 356 peptides that showed the strongest discrimination by IST are notable for not being significantly correlated to ELISA S/CO values (red dots above the white dashed line). This finding indicates that in addition to antibodies measured in both assays, unique antibody interactions were detected by IST. The 370 (356 + 14) library peptide sequences and associated raw signal data are tabulated in S2 Table.

**Fig 3:**
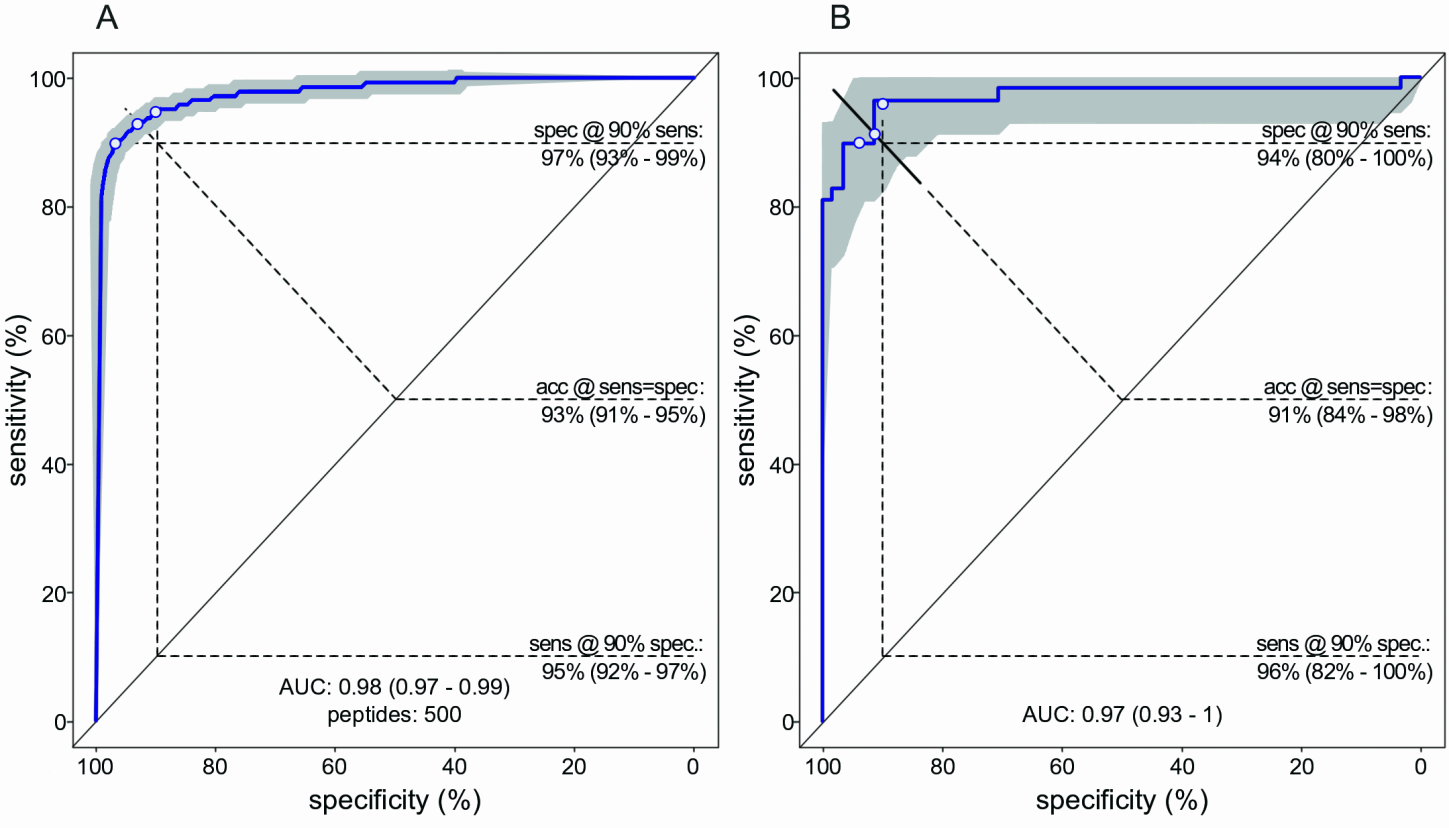
Performance of IST in distinguishing *T*. *cruzi* seropositive from seronegative donors. **(A)** Receiver Operating Characteristic (ROC) curve for the 2015 training cohort. The blue curve was generated by calculating the median of out-of-bag predictions in 100 four-fold cross-validation trials. **(B)** ROC curve for the 2016 verification cohort. The blue curve was generated by applying the training set-derived algorithm to predict the 2016 samples. Confidence intervals (CI), displayed in gray and numerically provided in parentheses, were estimated from the variation across cross-validation trials in the training cohort, and estimated by the DeLong method [53] in the verification cohort. sens = sensitivity, spec = specificity, acc = accuracy.

A support vector machine (SVM) classifier of Chagas positivity was developed in the 2015 cohort. The best average performance by cross-validation was achieved when the top 500 peptides, as ranked by Welch t-test, were input to the model. This number is greater than the 356 that met the Bonferroni significance cutoff, indicating that additional information was contained in some of the peptides that did not meet this stringent threshold. As few as 200 and as many as 4,000 library peptides could be used in the model with only modest reductions in performance (S1 Fig). Fig 3A shows the relationship between mean sensitivity and specificity of 100 iterations of five-fold cross validations, using these 500 top-scoring peptides as a function of diagnostic threshold. The AUC estimate of 0.98 means that any donor chosen randomly from within the 2015 cohort would have a 98% probability of being classified as seropositive if it was a blood-bank positive, and a 2% of being called positive if it was a blood-bank negative, with a 95% confidence interval (CI) of 97%-99%. At the threshold where sensitivity equaled specificity, the accuracy was 93% (CI = 91%-95%). The cross-validation estimates were confirmed by application of the 500 peptide SVM classifier developed with the 2015 cohort, to the independent 2016 cohort. The observed performance within this verification test set (AUC = 97%; accuracy = 91%) was within the 95% CI of the cross-validation estimates (Fig 3B).

The ROC curves take advantage of the continuous signal intensity values. Alternatively, a single, pre-specified positivity cut-off for Chagas positivity can be applied. Using 50% probability as an arbitrary diagnostic threshold to the predictions of the training set’s fixed model, performance metrics were calculated for the 2016 test set. Accuracy was determined to be 87% (95% CI = 81%-93%), sensitivity was 76% (95% CI = 63%-86%), specificity was 100% (95% CI = 94%-100%) and Cohen’s kappa was 76% (95% CI = 64%-87%). No test results were excluded. Table 1 presents the 2x2 matrix for these results. There were no false positives, and 14 false negatives. Of these false negatives, 8 had S/CO values of less than 2.0. A diagram of the sample IST testing flow and the blood bank’s (CTS) assignment of the same samples is provided in S2 Fig.

**Table 1:** Immune assay confusion matrix. Cross tabulation of the IST classifications and ELISA results of Chagas positivity for the 2016 test cohort.

		EIA	
		Negative	Positive
**IST**	**Negative**	58	14
	**Positive**	0	44

This same fixed classifier was used to assess the binding precision of the assay using a protocol in which a set of samples: four Chagas positive, three Chagas negative, and one control, was repeatedly assayed as described in the Methods section. These precision measurements are presented in Table 2. The values for inter-slide, inter-wafer, and inter-day are comparable to intra- and inter-assay CVs obtained with other *T*. *cruzi* ELISA tests. In addition to these precision studies, reproducibility of classification accuracy was determined across 22 different wafers using the QC sample layout described in the Methods; these analyses indicated AUCs >0.98 (median AUC = 1.0).

**Table 2:** Precision studies based on signal intensities of Chagas-informative peptides.

Measurement	CV (%)
Inter array	11.2
Inter slide	4.3
Inter wafer	2.7
Inter day	7.7
Inter batch	14.6

**Fig 4:**
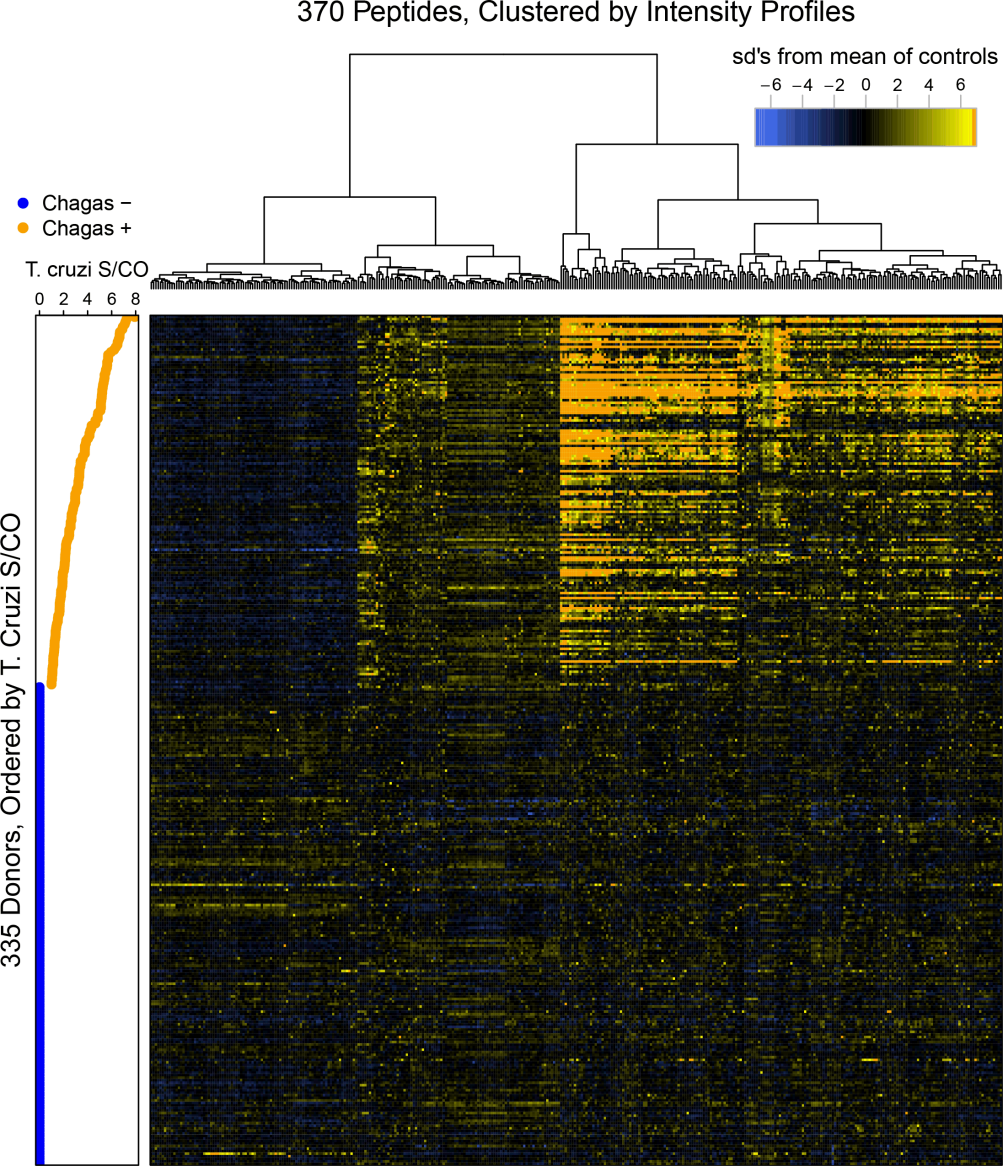
Signal intensity patterns displayed by donors’ Chagas sero-status informing peptides versus their S/CO value. A heatmap orders the ranges of signal intensities displayed by the 370 library peptides that inform a sample’s Chagas positivity status; the side-bar graph relates these to each donor’s ELISA S/CO value.

The results in Fig 4 explore the heterogeneity of antibody binding across the 2015 Chagas cohort. The relative signal intensities are displayed for the 370 (356 + 14) peptides described in Fig 2 that provided significant discrimination of Chagas positivity by t-test, by correlation to the ELISA S/CO levels or both criteria. In Fig 4, each peptide (x axis) for each donor (y axis) is represented, and is shaded relative to the difference in its intensity compared to the mean intensity of the same peptide in all seronegative donors, which serve as controls. The heatmap color scheme is scaled by the standard deviation (sd) of a feature’s signal from that of the seronegative controls. The legend has been truncated at 7 sd’s to permit smaller, but significant variations to be visualized. The donors were ordered by their median ELISA S/CO measurements, and these data are plotted along the left side of the heatmap. The peptides have been clustered as indicated by the dendrogram at the top. The distinction between ELISA seropositive and negative donors is evident as visualized in the IST heatmap, as are correlations within the ELISA seropositive samples to some peptides’ IST signal levels. The Chagas positive samples displayed at least three distinct binding profiles for a subset of the informative IST peptides: those with i) uniformly lower signal than seronegative samples, ii) marginally but uniformly higher signal than seronegative samples and iii) signal that increases as S/CO values increase. Peptide signal heterogeneity in the Chagas negative samples is relatively minor.

In addition to measuring IgG antibodies bound to the IST peptide array, IgA binding activity was measured by detecting plasma-antibody binding events with a fluorescently-labeled anti-IgA specific secondary reagent. Signal intensities of IgA binding events were generally lower than those of IgG binding, and fewer library peptides (224 versus 356) passed the Bonferroni cutoff for significantly different signal levels between the seropositive and negative donors. This can be visualized in a volcano plot (S3 Fig), in which the ratio of mean signal intensities between seropositive and negative samples was plotted versus the significance of the differential, for each peptide on the array. Within the 224 IgA-bound distinguishing peptides, 53 overlapped with those detected by probing with the anti-IgG secondary reagent (S4A Fig). The non-overlapping IgA peptide reactivities generally showed lower fold changes than that of the IgG reactivities. When measuring plasma IgA, all 23 peptides that met the Bonferroni threshold (p <4e-7) for correlation to *T*. *cruzi* S/CO values were among the 26 peptides correlated to S/CO when measuring IgG binding events. The effect sizes of the *T*. *cruzi* positive versus negative serogroups as measured by anti-IgA secondary were generally lower when compared to those measured by anti-IgG secondary. The peptides with the best effect sizes were predominantly those that overlapped between both detection methods, anti-IgG and anti-IgA secondary reagents (S4B Fig). The performance of the IgA classifier was strong, with a AUC of 0.94 and peak model size of 4,000 peptides (S5 Fig), though modestly less than that of the IgG classifier (AUC = 0.98). Combining the peptide lists for feature selection did not improve classifier performance (AUC = 0.98), suggesting some redundancy in the binding event information. Although the peak model size was 2,000 in the combined feature selection, whereas the IgG and IgA selection peaks were 500 and 4,000, respectively.

### Proteome-mapping the Chagas-informative peptides

**Fig 5:**
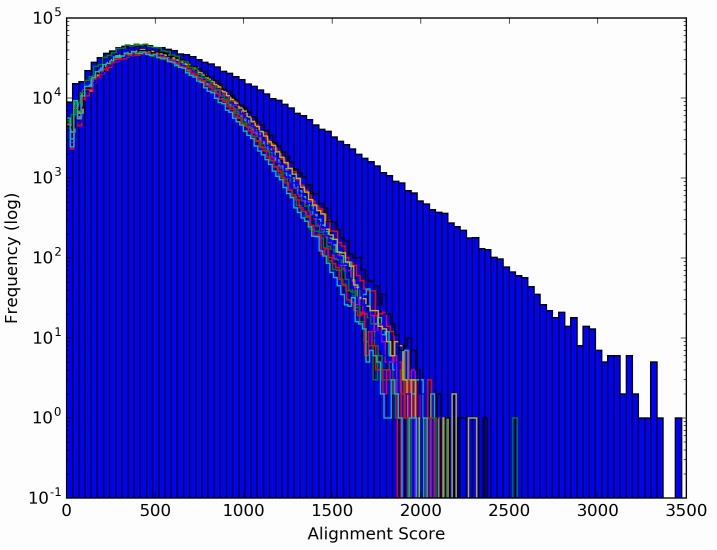
Alignment score frequencies displayed for the library peptides. A histogram of alignment scores from the top 370 informative peptides against all *T*. *cruzi* proteins are depicted in the blue bars. The mapping algorithm was repeated with equivalent alignments of 370 randomly chosen library peptides ten times. These yielded histograms that are shown as rainbow-colored line plots overlaid upon the blue bars.

The 356 library peptides that displayed significantly (p <4e-7) different signal intensities between Chagas positive and negative donors were combined with the 14 library peptides that were significantly correlated to S/CO values, but did not meet the Bonferroni cutoff for IST. Together, these 370 peptides were considered informative relative to Chagas positivity, and were used to explore possible informatic alignment to the *T*. *cruzi* proteome. A modified BLAST algorithm and scoring system was developed that used a sliding window of 20-mers (Methods). This yielded a ranked list of candidate protein regions (targeted 20-mers) shown in Table 3, with redundant hits stripped. The informative library peptides yielding these candidate *T*. *cruzi* proteome targets displayed alignment scores that greatly exceed the maximum scores obtained by performing the same analysis ten times with equally-sized (370) sets of peptides that were randomly selected from the library (Fig 5). For example, the maximum score obtained with the iterative sets of randomly selected peptides ranged from less than 2000 to a maximum of 2500; whereas the informative peptides generated maximum alignment scores from 3300 to 3500. Four of these top ten targets are protein regions from members of three families previously shown to be antigenic in Chagas patients. These are marked with asterisks in Table 3.

**Table 3:** Top ranking alignments of informative library peptides to the *T*. *cruzi* proteome.

	*T*. *cruzi* protein	Total Score	Target 20mer	# peptide hits	TrTrypDB Gene ID
1	*Mucin: TcMucII	3473	TRAPSRLREVDGSLGSSAWV	63	TcCLB.509753.300
2	Uncharacterized protein	3329	RRLSYRLRKREDGESYESYL	59	TcCLB.510575.140
3	Uncharacterized protein	3181	VPVLRVVDAEADERERNGAG	56	TCSYLVIO_001012
4	*Calmodulin	3096	TEVDVREALRVLDADGDGFL	57	TcCLB.510121.50
5	Uncharacterized protein	3091	SYRQQRYVDALRFALEEDHE	56	TcCLB.508269.50
6	*Mucin: TcMucII	3032	RAPSRLREFDGSLSSSAWVC	56	TcCLB.506741.110
7	Uncharacterized protein	3007	KLRQLDFVEDVLRKHPDKVE	52	TcCLB.507011.170
8	*Dispersed gene family protein 1 (DGF-1)	2983	HRGGLEALLRDGEDGDEDAQ	58	TcCLB.509735.84
9	Uncharacterized protein	2973	LPAQDSVVVRRLVDGQAPLR	51	TcCLB.506267.48
10	Vacuolar protein sorting-associated protein (Vps26)	2966	ERSPGLLGRLLRKVDGCDVR	52	TCSYLVIO_008174

* The families in which these proteins are members have been previously identified as antigenic

**Fig 6:**
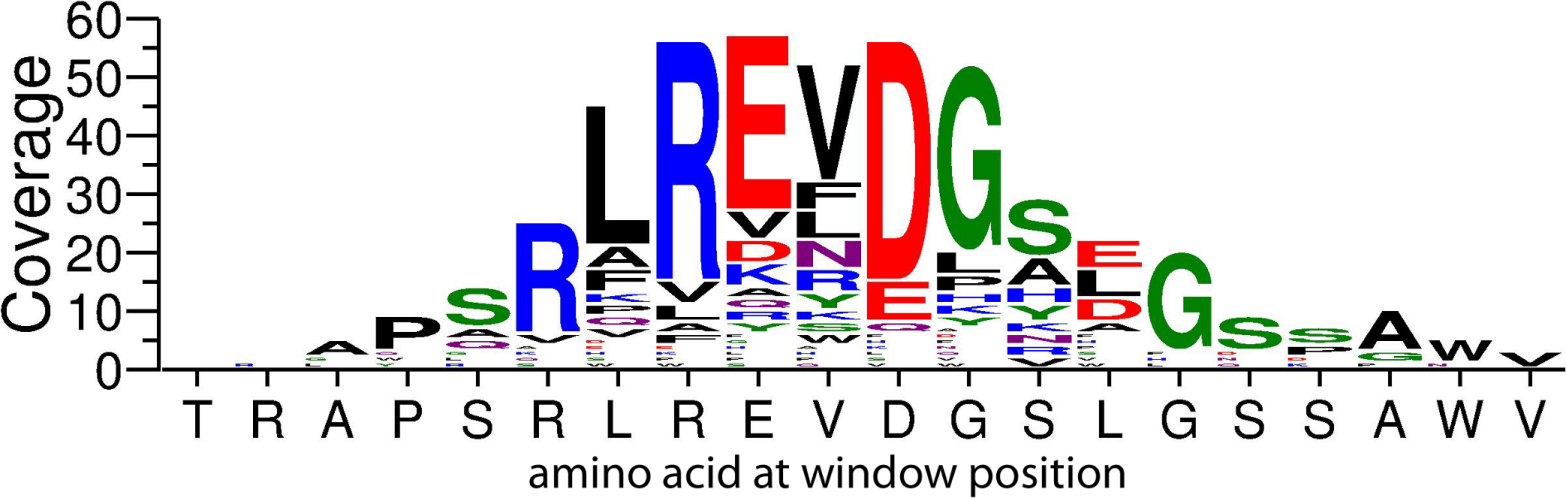
Representation of the levels of similarity of Chagas-informative peptides to a region within a subfamily of *T*. *cruzi* protein-antigens. Alignment of the 370 most Chagas-informative peptides to the Muc II GPI-attachment site is represented as a bar chart in which the bars have been replaced by the a.a. composition at each alignment position, using the standard single-letter codes. The x-axis indicates the conserved a.a. at the aligned position within mucin II. The y-axis indicates the coverage of that a.a. position by the classifying peptides. The total height of all letter-codes at a position corresponds to the absolute number of peptide alignments to that position. The proportional contribution of each a.a. to the letter-code bar is expressed by the height of each letter-code.

The top-scoring alignments of the Chagas informative peptides mapped to the C terminus of the mucin’s Muc II subfamily of surface glycoproteins. Only one protein ID is represented here; however, this C terminus is found with high sequence similarity in all 662 Muc family members (see Discussion). This library-peptide aligned region of Muc II includes a glycosylphosphatidylinositol (GPI) attachment site and corresponds to a highly immunogenic epitope found in Chagas patients [54]. The a.a.’s most frequently identified in the 63 aligned library peptides are summarized in Fig 6 as a modified WebLogo [55]. The corresponding *T*. *cruzi* sequence (TrTrypDB Gene ID = TcCLB.509753.300) is displayed along the x axis. Amino acid substitutions at any one position are shown vertically and the proportional coverage within the mapped library peptides is depicted by the height of the one-letter code. The total height of a letter-code bar indicates the absolute number of peptides aligning to the Muc II a.a. position. Another member of the Muc II protein subfamily is the sixth ranked target candidate, and it also maps to its C terminus. A member of a different *T*. *cruzi* surface glycoprotein family, the dispersed gene family proteins (DGF-1) [56], ranked eighth by the aligning algorithm. The library peptides mapped to its C-terminal region, which corresponds to the DGF-1 family’s consensus sequence that is shared by all 565 members. Other candidate targets showing high library-peptide alignment scores included proteins involved in calcium signal transduction (calmodulin), vesicle trafficking (vacuolar protein sorting-associated protein, Vps26) [57] or uncharacterized proteins. The ten top-ranked *T*. *cruzi* candidates, along with their subfamily and family members, were targeted by 222 of the aligned Chagas informative peptides. The modified WebLogos for each of the other 9 top proteome alignment targets is provided in S6 Fig.

### IST co-classification of Chagas positive donors and those positive for other blood panel diseases

A study was designed to determine whether IST could discriminate Chagas positive samples from samples of other infectious diseases. A subset of 88 *T*. *cruzi* seropositive samples from the full Chagas 2015 cohort was re-assayed, alongside 88 HBV, 71 HCV, and 88 WNV disease-positive plasma samples. The virus samples were assigned positivity by both indirect serologic and direct nucleic acid testing at CTS. All study samples were reported positive for only one of the four diseases. The demographic data showed a mix of genders and ethnicities, and a range of ages (S3 Table). Although demographic data are missing for many of the samples, there is a higher prevalence of Chagas positivity among Hispanic donors, consistent with disease prevalence in Central and South America. This higher prevalence was also seen within the full Chagas cohort (S1 Table). All IST assays for this study were performed on the same day and scanned immediately to acquire signal intensity measurements at each feature. The raw data was imported into R for analysis.

In a first analysis, a set of binary classifiers were developed to differentiate each of the four diseases from a combined class of the remaining three (Table 4). Performance metrics for each disease contrast and their corresponding 95% CI’s were determined by four-fold cross-validation analysis. The models generated similarly strong AUC’s, which ranged from 0.94 to 0.97, and corresponded to accuracies of 87%-92%. Nominally, the binary contrast of Chagas versus the combined class (Other) was best performing and HBV versus Other was the weakest; however, the parenthetically shown CI’s overlapped. While the number of optimal SVM input peptides for each contrast varied widely from 50 to 16,000 peptides, the number of peptides used did not significantly change performance. For instance, an SVM model size (k) of 50 for *T*. *cruzi* versus Other generated an AUC of 0.97; a model size of 4,000 generated an AUC of 0.96, indicating model size was robust.

**Table 4:** Binary classification of each of four disease classes versus a combined class of the remaining three.

Classes	AUC	Sensitivity@	Specificity@	Accuracy@	Model size
		90% spec^a^	90% sens^b^	spec^a^ = sens^b^	(k)
*T*. *cruzi* vs.	0.97	92%	94%	92%	50
Other	(0.96–0.98)	(90%-94%)	(90%-96%)	(90%-92%)	
HBV vs.	0.94	85%	85%	87%	16000
Other	(0.93–0.95)	(78%-90%)	(78%-90%)	(85%-90%)	
HCV vs.	0.96	90%	91%	90%	100
Other	(0.94–0.97)	(86%-94%)	(82%-96%)	(88%-93%)	
WNV vs.	0.96	87%	88%	89%	1000
Other	(0.95–0.97)	(78%-94%)	(84%-92%)	(86%-91%)	

^a^spec, specificity

^b^sens, sensitivity

In the next analysis, a model was developed to classify all four serologic states simultaneously, with one set of selected peptides (k = 75), and one algorithm. This multiclass model had marginally improved performance over the binary classifiers shown in Table 4. Namely, the four-fold cross validation analysis yielded multiclass AUC’s of 0.98 for Chagas, 0.96 for HBV, 0.95 for HCV, and 0.97 for WNV classifications. Table 5 presents the performance metrics of the assignments of each sample to a class based on its highest predicted probability. These probabilities assigned most samples to the same infectious disease class confirmed by CTS testing. In this confusion matrix, the performance of each binary contrast using the single multi-disease classifier is presented. The estimated overall multi-disease classification accuracy achieved 87%. Cohen’s unweighted kappa for the agreement between the true classes and the predictions of the multiclass model was calculated and determined to be 0.84 (95% CI, 0.79–0.89), indicating significantly greater concordance than expected by chance.

**Table 5:** Confusion matrix and performance estimates of the multiclass prediction model.

IST Classification	Blood Bank Confirmed Diagnosis				Performance Summary		
	*T*. *cruzi* pos	HBV pos	HCV pos	WNV pos	^a^Sens	^b^Spec	AUC
*T*. *cruzi*	77	3	1	2	93%	96%	0.98
HBV	3	79	12	2	82%	96%	0.96
HCV	0	3	55	2	92%	94%	0.95
WNV	8	3	3	82	85%	97%	0.97
Totals	88	88	71	88	Overall accuracy = 87%		

^a^Sens, sensitivity

^b^Spec, specificity

**Fig 7:**
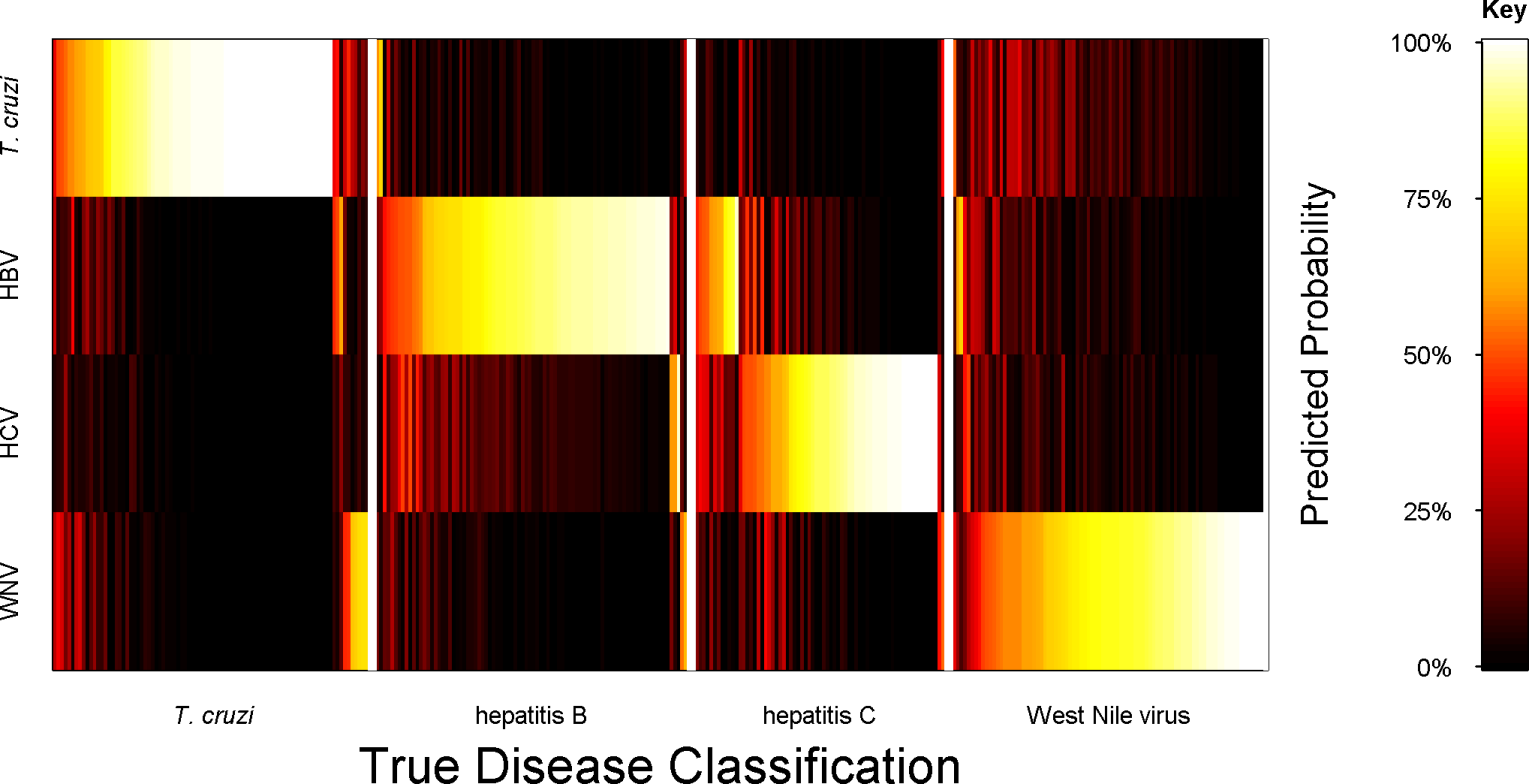
Probabilities of *T*. *cruzi*, hepatitis B, hepatitis C and West Nile virus class assignments. Mean probability assignments for each sample to each of the four disease classes were calculated by out-of-bag predictions from four-fold cross-validation analyses using a multiclass SVM machine classifier. The cross-validations were permutated and retested 100 times. Each sample had a predicted membership for each disease class that was given a color ranging from 0 (black) to 100% (white), corresponding to probabilities from 0% to 100%. A matrix arrangement was used to visualize all combinations of true (x axis) versus predicted (y axis) assignments.

A heat map is presented in Fig 7 that illustrates the mean probabilities of class membership for out-of-bag cross-validation predictions using the multiclass model (shown in Table 5). This was determined for each of the 335 study cohort samples, encompassing all four disease classes. The blood bank assignments are considered here as “true”, and the IST probabilities of being assigned to a disease class as “predicted”. The map presents the probabilities of each sample’s class assignment on a color scale from black to white; the samples are ordered along the x-axis by their true assignment. This enables the varying levels of IST-based distinction between classes to be visualized versus true classification. For example, prediction of true *T*. *cruzi* seropositive samples as *T*. *cruzi* versus each of the three viral diseases was strong, as the high probabilities (bright colors) in the correct class indicate. Even the few true *T*. *cruzi* samples with high probabilities for WNV prediction also displayed modest probabilities of a correct *T*. *cruzi* assignment. The predicted assignments of true HCV samples as HCV versus another liver virus, HBV, were weaker than those versus *T*. *cruzi* and WNV.

## Discussion

We have demonstrated the feasibility of using the ImmunoSignature Technology to detect *T*. *cruzi*-antibody positive individuals within a population of asymptomatic blood donors. The IST assignment of Chagas positivity closely matched that of the blood bank testing algorithms. A library of maximally-diverse peptides arrayed on a microchip were differentially bound by peripheral blood-antibodies in *T*. *cruzi* seropositive versus negative donors. These distinguishing peptides carried sequence-motifs with similarity to known immunogenic regions of several *T*. *cruzi* protein families, as well as several undescribed proteins that present here as possible antigens. In another study using the same library, both binary and simultaneous classifiers were developed that distinguished Chagas donors from those individuals who were positive but asymptomatic for three other blood panel tested diseases: hepatitis B, hepatitis C and West Nile virus.

These exploratory diagnostic studies were conducted on a well-characterized microarray platform. The use of photolithography and masks for the *in situ* process provides the opportunity for production-level scaling and reproducibility. The binding assay has a workflow analogous to an ELISA, and is similarly amenable to electronic tracing and robotic workstations.

The output from an IST binding assay is the quantified fluorescent measurements of antibody binding events that have occurred at every feature on an arrayed-library of peptides, which serve as epitope mimics. The diagnostic power derives from the identification of binding profiles, based on numerous selective antibody binding events, that are consistent within a disease class and consistently different relative to another. Notably, the same library design can be used to identify peptides that distinguish any disease class or health contrast, and a single assay can be used to simultaneously detect multiple serologic states.

While we have optimized array manufacturing and assay conditions, we recognize that alternative library designs may provide improved overall performance or improvements for a particular diagnostic application. For example, increasing the number of peptides in the library will enable denser sampling of sequence space but would require either reducing the number of arrays manufactured per wafer or reducing feature size. Increasing the length of the library-peptides may enable longer linear and conformational epitopes to be mimicked; on the other hand, it may enable two or more antibodies with distinct recognition sites to find a mimic on the same peptide and thereby confuse interpretation of the collective signal. Our exploration of these parameters is in progress.

The first Chagas study demonstrated that signal intensities of some of the library peptides which significantly distinguished the *T*. *cruzi* seropositive from seronegative disease states were also significantly correlated to ELISA-derived S/CO values. This finding provides an orthogonal verification of the IST test results relative to the current diagnostic standards. This finding also suggests that similar antibodies are being detected by the two different test platforms. However, there were many array binding events that did not correlate with the donor’s S/CO value yet were determined to differ significantly between classes and strengthened the IST classifier. This suggests that in addition to commonly measured antibodies, there are also unique antibody-binding events measured only by the IST platform. While beyond the scope of the work presented here, these unique events hold the potential for contributing to further assessments of *T*. *cruzi*-elicited immune activity. Next steps will be to investigate this possibility relative to clinically important stratifications of *T*. *cruzi*-related disease states.

The peptides comprising the distinctive and consistent binding profiles of Chagas positive versus negative donors in the 2015 cohort were input to a machine learning algorithm to yield an estimated performance. A classifier was fixed, then verified by using it to predict the Chagas positivity status of an independent 2016 cohort of plasma samples. The accuracy of the IST assay evaluated here approached but was modestly reduced compared to that of the ELISA standard, as calculated in a high risk donor population trial [58] (sensitivity = specificity at 91% versus 97%). However, since the ELISA was used to designate the “true” serologic class assignment, we cannot determine from this work whether the IST method was either more or less accurate than the ELISA. In an alternative analysis of the SVM classification results, a pre-specified cut-off of positivity was applied to each of the samples in the 2016 test cohort. Samples with a predicted Chagas probability of greater than 0.5 were classified as positive; those with probabilities of less than 0.5 were classified as negative. Cross tabulation of these assignments with the blood banks revealed zero false positives. The 14 differential assignments were all IST false negatives. We suggest that this result indicates either a lower IST sensitivity, or its higher specificity. This will be explored in future work using longitudinally collected samples from *T*. *cruzi* seropositive individuals with ultimately different clinical outcomes.

The IST method may hold immune response information not extractable from current tests. To address this hypothesis, a high-resolution view of a deeper structure within the class-specific binding profiles was attempted. Within the set of peptides that were found to bind with significantly different intensities to the Chagas positive versus Chagas negative donor samples, intragroup heterogeneity was observed. For example, there were peptides that displayed low intensities and another set that showed high signal intensities versus the Chagas negative donors. In addition to these two *T*. *cruzi* seropositive class clusters of either uniformly low or high signal, about half of the peptides that distinguished the positive class from the negative displayed a range of signal intensity that increased with increasing S/CO values. These are the S/CO correlated peptides. There is a sub-cluster within these correlated peptides that increase gradually while another appears to transition to higher signal more suddenly at an S/CO of ~4.0. Further work will be needed to determine whether there is any clinical significance to these different clusters, such as possibly indicating infection status or likelihood of progression to symptomatic disease [52].

As another means to potentially capture additional information about the *T*. *cruzi-*elicited antibody response, binding signals were detected using an anti-IgA, instead of anti-IgG, secondary antibody for detection. Given the mucosal route of parasite entry and evidence of protective mucosal immune responses to Chagas [59, 60], differential binding activity was anticipated and indeed found. However, no additional disease classifying peptides were identified in these interactions, suggesting that the same *T*. *cruzi* epitopes dominate both antibody isotype responses.

An alignment strategy was attempted to explore whether the class-distinguishing peptides could be mapped to any *T*. *cruzi* proteome targets. The diverse sequence design of the peptides in our arrayed library is directed at using mimotopes of true epitopes to broadly sample antibody reactivities, without requiring knowledge of the antigen responsible. However, we hypothesized that some of these mimotopes might sufficiently resemble a previously characterized linear epitope to be identified by an alignment algorithm. This was a further challenge as *T*. *cruzi* genome harbors 12,000 genes, half of which carry repetitive sequences and mainly comprise large multigene families and retrotransposons [61]. Although overall synteny between *T*. *cruzi* and its most-related kinetoplastid, *Leishmania major*, is 75%, species-specific islands have been identified within the encoded surface glycoproteins [34]. These glycoproteins are suggested to be diagnostically important as cross-reactivity, especially with *Leishmania* spp., is a significant source of false positive diagnosis in co-endemic regions [62]. In our analysis, the top ten scoring targets included the *T*. *cruzi* mucin (Muc II) and DGF-1 surface glycoprotein families. These linear peptidic hits support the interpretation that the IST assay is truly measuring *T*. *cruzi* antibody binding events, and leave open the possibility that the other distinguishing but unmapped peptides may mimic non-linear or non-peptidic ligands such as structural or glycosyl moieties. Alternatively, the unmapped peptides that differentially bound Chagas positive plasma may be mimicking non-parasitic self-antigens. While controversial, autoimmunity has been hypothesized as contributing to Chagas disease [63].

Mucins (Muc I and Muc II) comprise the third largest protein family of the *T*. *cruzi* proteome, holding 662 members, which together make up the protective glycoconjugate coat that covers the parasite surface. All Muc proteins contain an N terminal signal sequence for secretory pathway targeting, a C terminal GPI anchor attachment site, and a central region that is hypervariable (HV) [64]. The central domain of variable sequences stimulates a myriad of immune responses that lead to T cell anergy. By contrast, the GPI anchor region is both conserved and consistently immunogenic [34, 54]. The highest scoring IST peptide-motif alignments mapped to the C terminus of the Muc II protein subfamily. Although this region is nearly identical in all members, another Muc II family member with a variant C terminus ranked sixth. A mucin-like glycoprotein called tryptomastigote small surface antigen (TSSA) has been reported to elicit strong antibody responses [65] yet was not targeted by our best aligning peptides. It is possible that it was missed because the combinatorial library used here was not designed for proteome mapping, even though we explored it. Alternatively, it is because TSSA is expressed only by the infective, bloodstream tryptomastigotes [66]. The samples analyzed here were from asymptomatic donors; these people were likely to be harboring intracellular amastigotes, associated with the chronic infection.

The DGF-1’s are the fifth largest protein family with 565 N-glycosylated members [56]. Their conserved adhesion motifs suggest that they function similarly to integrins, and may play roles in extracellular matrix interactions and bidirectional signal transduction [67]. The consensus C terminus of the DGF-1 protein family ranked eighth in alignments by the IST peptide motifs. Members of two much smaller protein families, also involved in signal transduction and protein trafficking, were alignment targets: calmodulin and vacuolar protein sorting-associated protein 26 (Vps26). *T*. *cruzi* and human calmodulins display a high degree of similarity; the IST classifying peptides that aligned to *T*. *cruzi* calmodulin also aligned to the human protein. Human calcium/calmodulin-dependent protein kinase is known to play a critical role in cardiomyocyte survival and cardiomyopathy [68–70]. Finally, the identification of Vps26 is noteworthy as the protozoan transforms from tryptomastigote to amastigote within the vacuole of an infected host [10]. Together these protein families, collectively well over 1200 proteins, could account for 222 of the 370 mimetic peptides displaying highly significant, Chagas-distinguishing binding signals. While this represents a large portion of the antibody profile, there remains 148 mimetic responses to be explored.

None of these protein families are currently in any purified antigen-based Chagas test. However, a large project conducted several years ago started with 400 recombinant Chagas proteins and identified 16 candidates in a bead-based screening assay for serologic reactivity [71]. Both a calmodulin and a DGF-1 protein were found to be consistently reactive in *T*. *cruzi* seropositive Chagas patients. However, full length or large protein fragments were used such that the epitopes within these targets were not defined for our comparison here. An earlier mentioned antigen discovery study used a tiled peptide array spanning known or predicted *T*. *cruzi* antigens to probe purified IgG’s from nine *T*. *cruzi* seropositive individuals [33]. Their results showed positive binding to peptides corresponding to the same region of the Muc II consensus sequence that we identified here. We suggest that a Chagas proteome-targeted array holds great promise for new *T*. *cruzi* antigen discovery and that the IST mimotope array may be complimentary in providing additional *T*. *cruzi-*epitope screening capacity. However, IST also provides a single technology for developing a scalable, diagnostic platform applicable to Chagas, in addition to other diseases or disease stratifications.

The finding that a single test algorithm could distinguish Chagas positivity from that of several other blood panel diseases supports the interpretation that disease-specific antibody binding profiles can be identified on the mimetic peptide arrays. It is possible that a general state of immune activation such as inflammation might also be detected on the arrays, perhaps indirectly by some general change in IgG binding activity. However, any such antibody-bound peptides would not be anticipated to specifically distinguish one disease state from another and therefore would not be identified in the analysis. IST disease specificity was suggested by early studies with small cohorts (n = 10) in which different sets of peptides were identified that bound serum samples from patients with an infectious disease or cancer [39, 40]. Shown here, the multi-disease classifier of four blood panel diseases suggests that a single IST test, with a single set of peptides, might be developed for the detection of a panel of diseases. The ability to successfully co-classify diseases also suggests that IST may provide an opportunity for building a diagnostic with reduced cross-reactivity. The larger number of biomarker ligands may provide the improved specificity. Clinically relevant disease contrasts to assess next on this platform will be *T*. *cruzi* versus parasitic infections endemically found in the same regions such as *Leishmania* spp. [72] and *Plasmodium* spp [73], and the nonpathogenic *T*. *rangeli* [74].

Whereas blood banks test samples from asymptomatic individuals, clinical settings require the diagnosis of both asymptomatic and symptomatic patients, in acute or chronic stages of disease. Evaluating the potential for IST to contribute in these contexts will require ongoing sample collections and experimentation. The subclasses of binding profiles within the Chagas positive group may contain information that we have not yet learned to extract or interpret. With the relevant sample cohorts and clinical annotation for training and testing, this might be achieved.

While the express purpose in the presented work was not to discover new *T*. *cruzi* antigens or drug targets, or to develop a diagnostic for use in Chagas endemic areas, the results direct attention to these potential applications. Additional work will be required to assess the capability of the IST for addressing these clinical needs. Relative to the main objective of this work, the blood donor studies presented here show that IST is a viable tool for exploring the complexity of human immune responses to *T*. *cruzi*, and have highlighted platform-specific attributes that may hold advantages for Chagas diagnostics, prognostication, and monitoring of disease progression or resolution.

## Supporting information

S1 Fig Measuring IgG binding events by IST distinguishes *T. cruzi* seropositive from seronegative donors. Performance as assessed by AUCs is plotted versus SVM input model size (k).
(TIF)

S2 Fig Diagram presenting the sample testing flow of the 2016 Chagas study cohort. This was the ImmmunoSignature test verification set for *T*. *cruzi* seropositivity.
(PDF)

S3 Fig A set of library peptides reproducibly displayed IgA antibody-binding signals that were significantly different between Chagas seropositive and seronegative donors. A volcano plot is used to assess this discrimination as the joint distribution of t-test *p*-values versus the ratio of geometric mean intensities for Chagas positive donors relative to Chagas negative. The density of peptides at each plotted position is indicated by the color scale. The 224 peptides above the dashed white line discriminate between positive and negative seropositivity by IST with 95% confidence after applying a Bonferroni adjustment for multiplicity. The colored circles indicate individual peptides with intensities that were significantly correlated to the *T*. *cruzi* ELISA-derived signal over cutoff (S/CO) value either by a Bonferroni threshold of *p* < 4e-7 (green) or the less stringent false discovery rate (FDR) of <10% (blue).
(TIF)

S4 Fig Comparison of IgG versus IgA-binding peptides that distinguish Chagas positive from negative groups. **(A)** The number of unique and overlapping peptides bound by anti-IgA versus anti-IgG secondary antibodies are displayed as a Venn diagram. **(B)** The effect sizes of the IgG binding events are plotted against IgA. Coloring coding matches in (A) and (B): plotted peptides bound differentially by IgG-only are blue, IgA-only are red, and those differentially bound by both IgG and IgA are purple.
(TIF)

S5 Fig Measuring IgA binding events by IST distinguishes *T. cruzi* seropositive from seronegative donors. Performance as assessed by AUCs is plotted versus SVM input model size (k).
(TIF)

S6 Fig WebLogo representations of the similarity of Chagas-informative peptides to other *T. cruzi* proteins. The alignments of the 370 most Chagas-informative peptides to additional *T*. *cruzi* targets, named and ranked 2–10 in Table 3, are represented as bar charts in which the bars have been replaced by the a.a. composition at each alignment position, using the standard single-letter codes. The x-axes indicate the conserved a.a. at the aligned position within the targeted proteins. The y-axes indicate coverage of that a.a. position by the classifying peptides. The total height of all letter-codes at a position corresponds to the absolute number of peptide alignments to that position. The proportional contribution of each a.a. to the letter-code bar is expressed by the height of each letter-code.
(TIF)

S1 Table Description of donors in the Chagas study. (PDF)

S2 Table List of Chagas sero-status informing peptides. These are the 370 peptides that displayed signal intensities that significantly discriminated Chagas positive (+) from negative (-) donors, and/or are correlated to median *T*. *cruzi* S/CO measurements.
(XLSX)

S3 Table Description of donors in the multi-disease study. (PDF)

S4 Table STARD 15 checklist for reporting of studies of diagnostic accuracy. (PDF)
