# Correction: An ImmunoSignature test distinguishes *Trypanosoma cruzi*, hepatitis B, hepatitis C and West Nile virus seropositivity among asymptomatic blood donors

**DOI:** 10.1371/journal.pntd.0006228

**Published:** 2018-02-01

**Authors:** The PLOS Neglected Tropical Diseases Staff

There are errors in Tables 3–5 which arose during typesetting. The publisher apologizes for these errors.

**Table 3:** Top ranking alignments of informative library peptides to the *T*. *cruzi* proteome.

	*T*. *cruzi* protein	Total Score	Target 20mer	# peptide hits	TrTrypDB Gene ID
1	Mucin: TcMucII*	3473	TRAPSRLREVDGSLGSSAWV	63	TcCLB.509753.300
2	Uncharacterized protein	3329	RRLSYRLRKREDGESYESYL	59	TcCLB.510575.140
3	Uncharacterized protein	3181	VPVLRVVDAEADERERNGAG	56	TCSYLVIO_001012
4	Calmodulin*	3096	TEVDVREALRVLDADGDGFL	57	TcCLB.510121.50
5	Uncharacterized protein	3091	SYRQQRYVDALRFALEEDHE	56	TcCLB.508269.50
6	Mucin: TcMucII*	3032	RAPSRLREFDGSLSSSAWVC	56	TcCLB.506741.110
7	Uncharacterized protein	3007	KLRQLDFVEDVLRKHPDKVE	52	TcCLB.507011.170
8	Dispersed gene family protein 1 (DGF-1)*	2983	HRGGLEALLRDGEDGDEDAQ	58	TcCLB.509735.84
9	Uncharacterized protein	2973	LPAQDSVVVRRLVDGQAPLR	51	TcCLB.506267.48
10	Vacuolar protein sorting-associated protein (Vps26)	2966	ERSPGLLGRLLRKVDGCDVR	52	TCSYLVIO_008174

* The families in which these proteins are members have been previously identified as antigenic

**Table 4:** Binary classification of each of four disease classes versus a combined class of the remaining three.

Classes	AUC	Sensitivity@ 90% spec^a^	Specificity@ 90% sens^b^	Accuracy@ spec^a^=sens^b^	Model size (k)
*T*. *cruzi* vs. Other	0.97 (0.96-0.98)	92% (90%-94%)	94% (90%-96%)	92% (90%-92%)	50
HBV vs. Other	0.94 (0.93-0.95)	85% (78%-90%)	85% (78%-90%)	87% (85%-90%)	16000
HCV vs. Other	0.96 (0.94-0.97)	90% (86%-94%)	91% (82%-96%)	90% (88%-93%)	100
WNV vs. Other	0.96 (0.95-0.97)	87% (78%-94%)	88% (84%-92%)	89% (86%-91%)	1000

^a^spec, specificity

^b^sens, sensitivity

**Table 5:** Confusion matrix and performance estimates of the multiclass prediction model.

IST Classification	Blood Bank Confirmed Diagnosis				Performance Summary		
	*T*. *cruzi* +	HBV+	HCV+	WNV+	Sens^a^	Spec^b^	AUC
*T*. *cruzi*+	77	3	1	2	93%	96%	0.98
HBV+	3	79	12	2	82%	96%	0.96
HCV+	0	3	55	2	92%	94%	0.95
WNV+	8	3	3	82	85%	97%	0.97
Totals	88	88	71	88	Overall accuracy = 87%		

^a^Sens, sensitivity

^b^Spec, specificity
