# Supplementary figures and images for: An ImmunoSignature test distinguishes Trypanosoma cruzi, hepatitis B, hepatitis C and West Nile virus seropositivity among asymptomatic blood donors
Source: PLoS Negl Trop Dis. 2017 Sep 5;11(9):e0005882. doi: 10.1371/journal.pntd.0005882 (PMC5600393; doi:10.1371/journal.pntd.0005882)

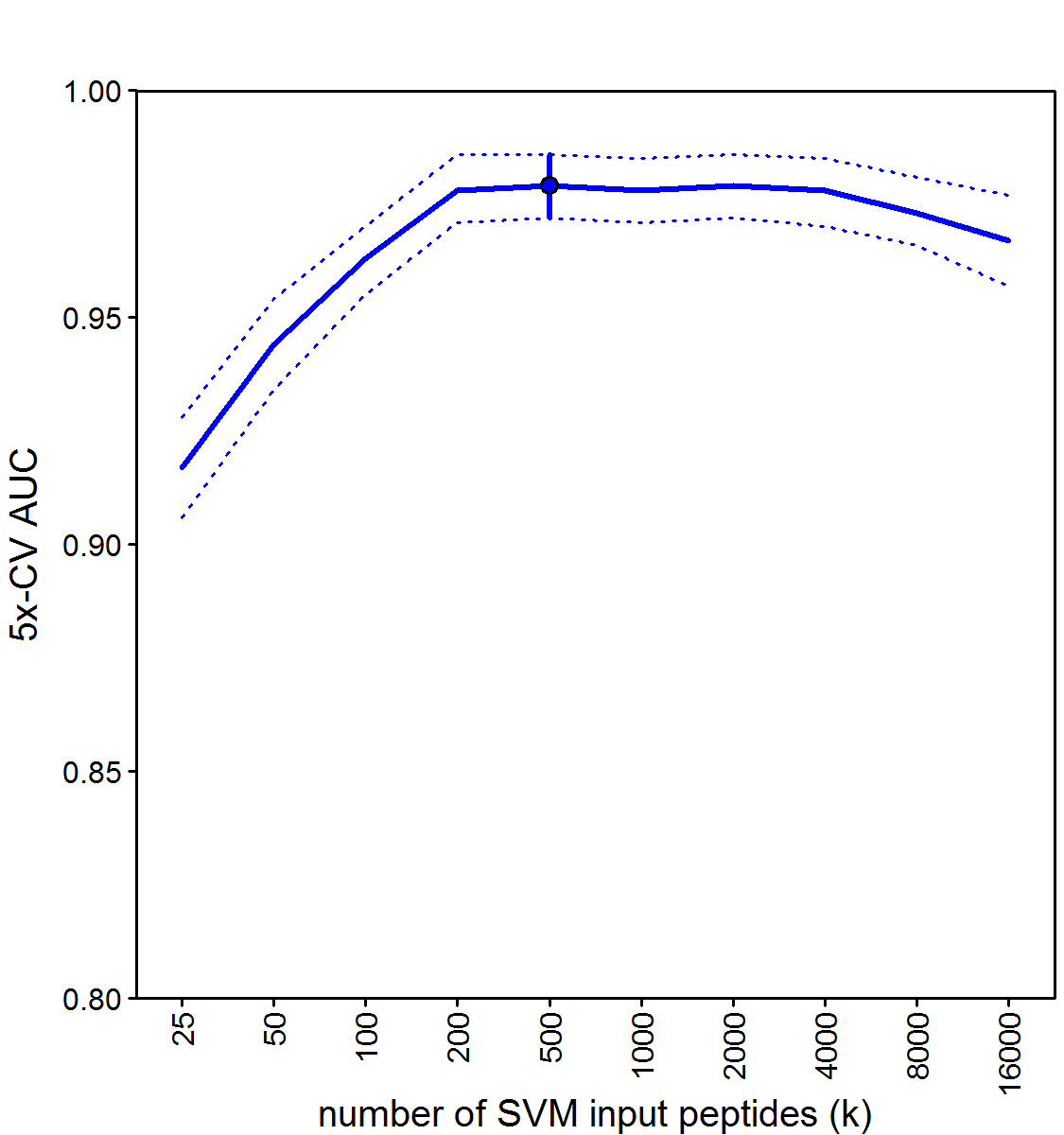

Supplement: S1 Fig — Performance as assessed by AUCs is plotted versus SVM input model size (k). (TIF) [file pntd.0005882.s001.tif]

**Index test: IST fixed classifier**

**Reference test: CTS algorithm  
(3x EIA, RIPA)**

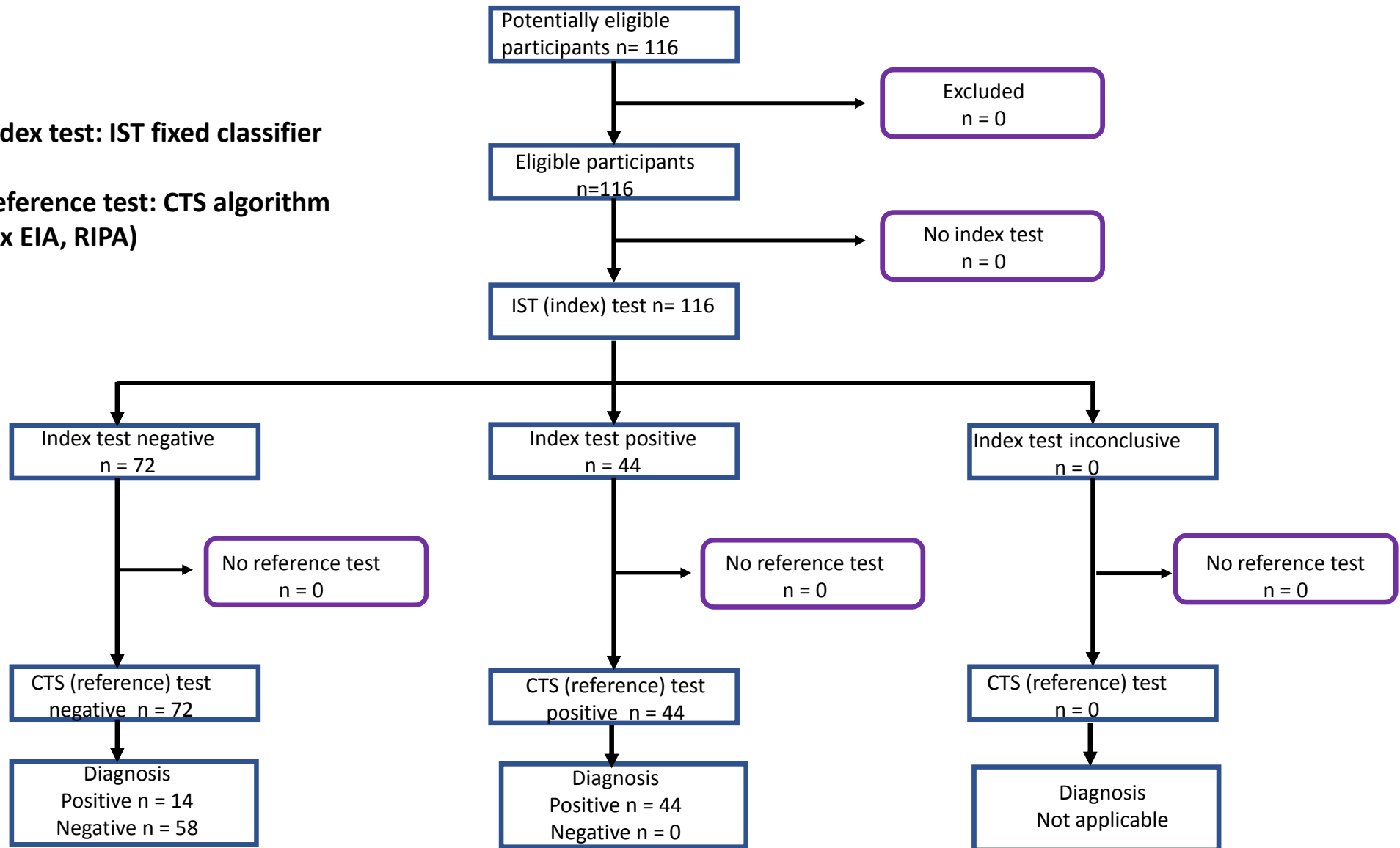

Supplement: S2 Fig — This was the ImmmunoSignature test verification set for T. cruzi seropositivity. (PDF) [file pntd.0005882.s002.pdf]

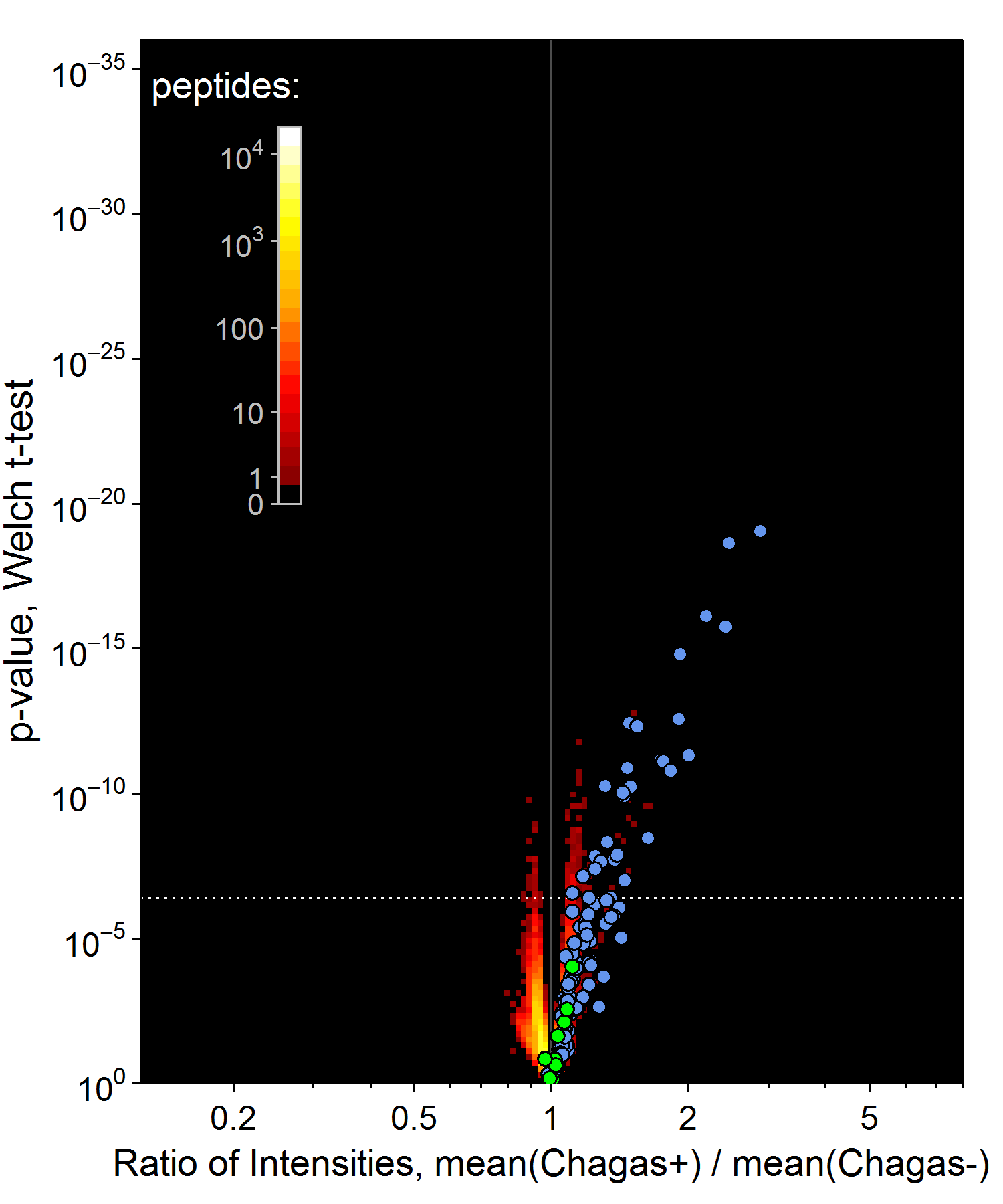

Supplement: S3 Fig — A volcano plot is used to assess this discrimination as the joint distribution of t-test p-values versus the ratio of geometric mean intensities for Chagas positive donors relative to Chagas negative. The density of peptides at each plotted position is indicated by the color scale. The 224 peptides above the dashed white line discriminate between positive and negative seropositivity by IST with 95% confidence after applying a Bonferroni adjustment for multiplicity. The colored circles indicate individual peptides with intensities that were significantly correlated to the T. cruzi ELISA-derived signal over cutoff (S/CO) value either by a Bonferroni threshold of p < 4e-7 (green) or the less stringent false discovery rate (FDR) of <10% (blue). (TIF) [file pntd.0005882.s003.tif]

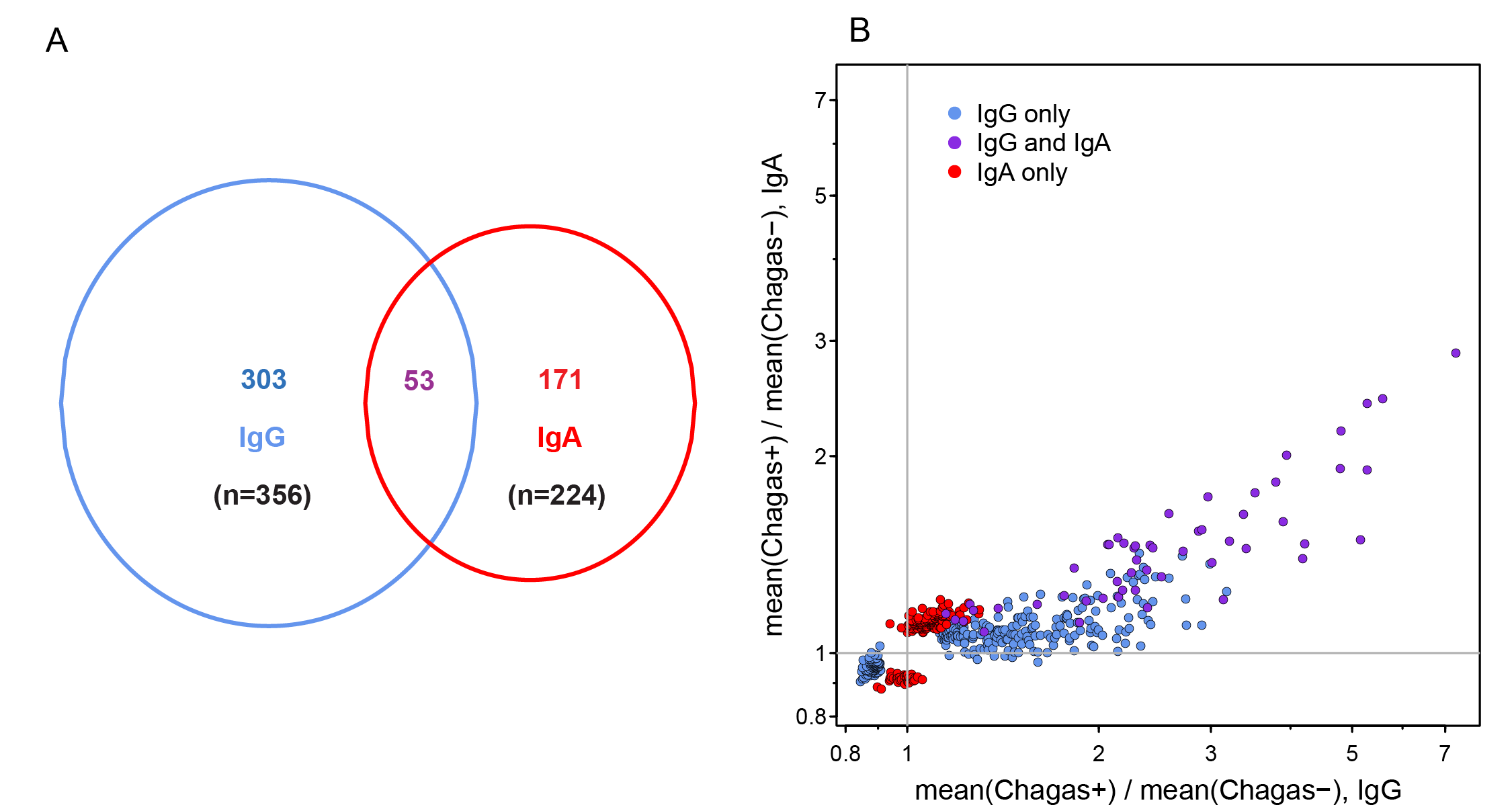

Supplement: S4 Fig — (A) The number of unique and overlapping peptides bound by anti-IgA versus anti-IgG secondary antibodies are displayed as a Venn diagram. (B) The effect sizes of the IgG binding events are plotted against IgA. Coloring coding matches in (A) and (B): plotted peptides bound differentially by IgG-only are blue, IgA-only are red, and those differentially bound by both IgG and IgA are purple. (TIF) [file pntd.0005882.s004.tif]

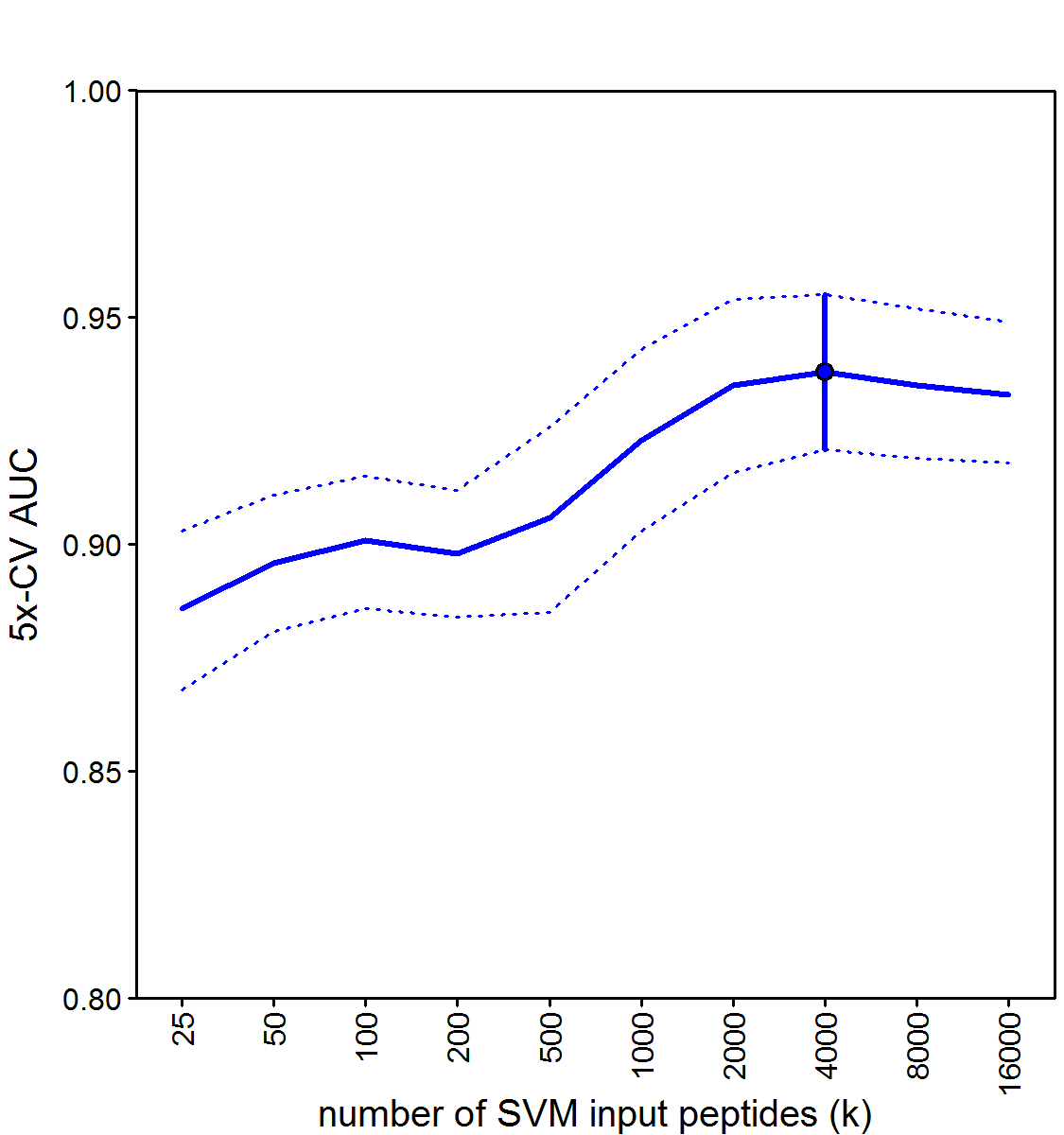

Supplement: S5 Fig — Performance as assessed by AUCs is plotted versus SVM input model size (k). (TIF) [file pntd.0005882.s005.tif]

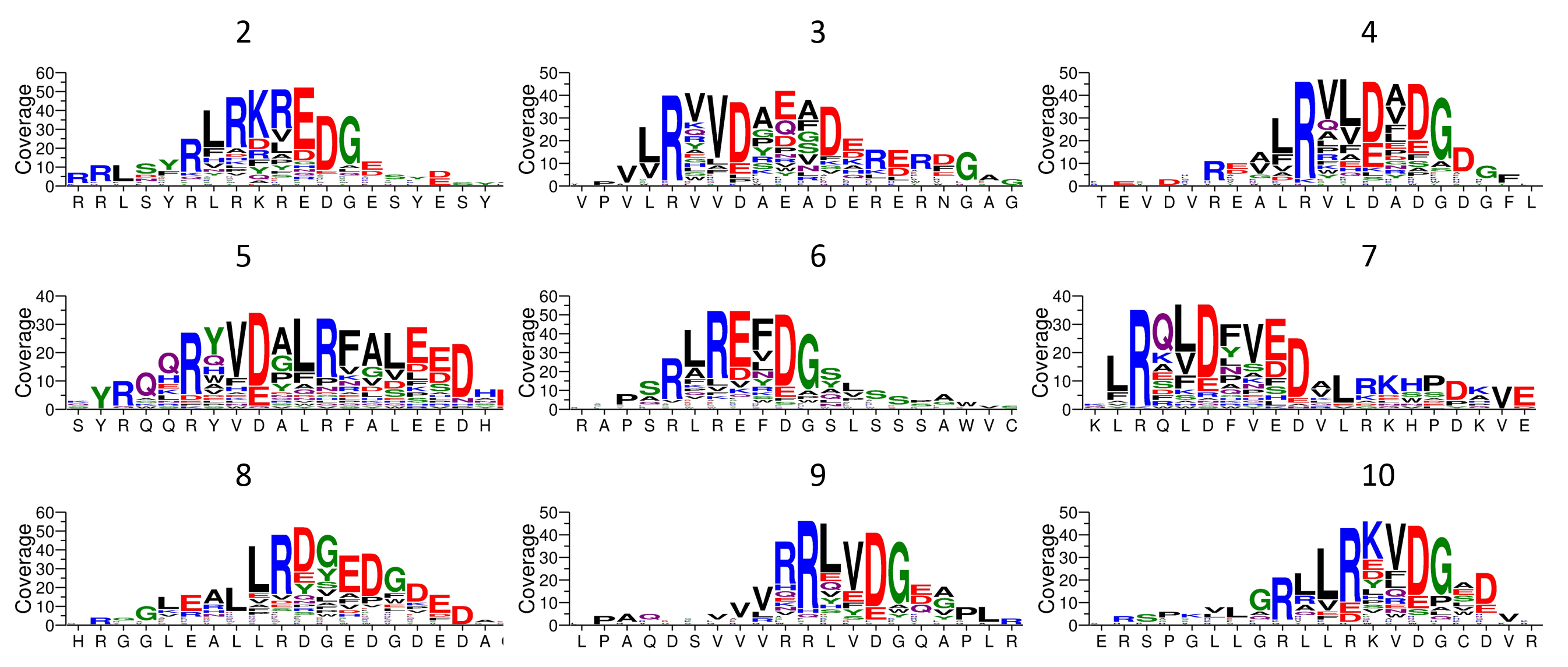

Supplement: S6 Fig — The alignments of the 370 most Chagas-informative peptides to additional T. cruzi targets, named and ranked 2–10 in Table 3, are represented as bar charts in which the bars have been replaced by the a.a. composition at each alignment position, using the standard single-letter codes. The x-axes indicate the conserved a.a. at the aligned position within the targeted proteins. The y-axes indicate coverage of that a.a. position by the classifying peptides. The total height of all letter-codes at a position corresponds to the absolute number of peptide alignments to that position. The proportional contribution of each a.a. to the letter-code bar is expressed by the height of each letter-code. (TIF) [file pntd.0005882.s006.tif]
