# Supplementary material for: An ImmunoSignature test distinguishes Trypanosoma cruzi, hepatitis B, hepatitis C and West Nile virus seropositivity among asymptomatic blood donors
Source: PLoS Negl Trop Dis. 2017 Sep 5;11(9):e0005882. doi: 10.1371/journal.pntd.0005882 (PMC5600393; doi:10.1371/journal.pntd.0005882)
Supplement: S1 Table — (PDF) [file pntd.0005882.s007.pdf]

S1 Table. Description of donors in the Chagas only study.

	Training cohort (2015)					Test cohort (2016)			
	all	*T. cruzi* neg	*T. cruzi* pos	S/CO >4		all	*T. cruzi* neg	*T. cruzi* pos	S/CO >4
Group size	335	189	146	49		116	58	58	31
Gender									
female	90	80	10	2		48	24	24	11
male	127	109	18	6		68	34	34	20
unknown	118	0	118	41		0	0	0	0
Ethnicity									
white	145	144	1	1		14	8	6	4
Hispanic	49	32	17	4		84	43	41	24
black	4	4	0	0		3	2	1	0
other	10	9	1	0		2	2	0	0
unknown	127	0	127	44		13	3	10	3
Age bin									
(15-20)	10	9	1	1		16	7	9	5
(20-30)	29	26	3	0		20	11	9	5
(30-40)	52	46	6	1		24	14	10	6
(40-50)	38	33	5	2		26	9	17	7
(50-60)	38	32	6	1		21	11	10	7
(60-70)	29	26	3	2		7	4	3	1
(70-87)	21	17	4	1		2	2	0	0
unknown	118	0	118	41		0	0	0	0
