# Supplementary material for: An ImmunoSignature test distinguishes Trypanosoma cruzi, hepatitis B, hepatitis C and West Nile virus seropositivity among asymptomatic blood donors
Source: PLoS Negl Trop Dis. 2017 Sep 5;11(9):e0005882. doi: 10.1371/journal.pntd.0005882 (PMC5600393; doi:10.1371/journal.pntd.0005882)
Supplement: S3 Table — (PDF) [file pntd.0005882.s009.pdf]

S3 Table. Description of donors in the multi-disease study.

	all	*T. cruzi*	HBV	HCV	WNV
Group size (n)	335	88	88	71	88
Gender					
female	62	27	7	7	21
male	102	30	11	21	40
unknown	171	31	70	43	27
Ethnicity					
white	70	5	2	16	47
Hispanic	54	38	1	5	10
black	5	0	4	1	0
other	18	4	11	2	1
unknown	188	41	70	47	30
Age bin					
(16-20)	11	3	3	1	4
(20-30)	30	7	6	7	10
(30-40)	26	14	2	2	8
(40-50)	36	11	3	6	16
(50-60)	35	12	1	10	12
(60-70)	18	6	3	2	7
(70-87)	8	4	0	0	4
unknown	171	31	70	43	27
