# Supplementary material for: An ImmunoSignature test distinguishes Trypanosoma cruzi, hepatitis B, hepatitis C and West Nile virus seropositivity among asymptomatic blood donors
Source: PLoS Negl Trop Dis. 2017 Sep 5;11(9):e0005882. doi: 10.1371/journal.pntd.0005882 (PMC5600393; doi:10.1371/journal.pntd.0005882)
Supplement: S4 Table — (PDF) [file pntd.0005882.s010.pdf]

S4 Table. STARD 15 checklist for reporting of studies of diagnostic accuracy.

Section & Topic	No	Item	Reported on page #
**TITLE OR ABSTRACT**			
	**1**	Identification as a study of diagnostic accuracy using at least one measure of accuracy<br>(such as sensitivity, specificity, predictive values, or AUC)	1-2 (Abstract)
**ABSTRACT**			
	**2**	Structured summary of study design, methods, results, and conclusions<br>(for specific guidance, see STARD for Abstracts)	1-2
**INTRODUCTION**			
	**3**	Scientific and clinical background, including the intended use and clinical role of the index test	2-4
	**4**	Study objectives and hypotheses	1, 4
**METHODS**			
Study design	**5**	Whether data collection was planned before the index test and reference standard were performed (prospective study) or after (retrospective study)	4-5<br>Retrospective study
Participants	**6**	Eligibility criteria	4-5, 10-11
	**7**	On what basis potentially eligible participants were identified<br>(such as symptoms, results from previous tests, inclusion in registry)	4-5
	**8**	Where and when potentially eligible participants were identified (setting, location and dates)	4-5
	**9**	Whether participants formed a consecutive, random or convenience series	4-5
Test methods	**10a**	Index test, in sufficient detail to allow replication	5-8
	**10b**	Reference standard, in sufficient detail to allow replication	4-5
	**11**	Rationale for choosing the reference standard (if alternatives exist)	3
	**12a**	Definition of and rationale for test positivity cut-offs or result categories of the index test, distinguishing pre-specified from exploratory	11-14
	**12b**	Definition of and rationale for test positivity cut-offs or result categories of the reference standard, distinguishing pre-specified from exploratory	11-13
	**13a**	Whether clinical information and reference standard results were available to the performers/readers of the index test	8
	**13b**	Whether clinical information and index test results were available to the assessors of the reference standard	8
Analysis	**14**	Methods for estimating or comparing measures of diagnostic accuracy	11-14
	**15**	How indeterminate index test or reference standard results were handled	13-14, no indeterminates
	**16**	How missing data on the index test and reference standard were handled	Not applicable
	**17**	Any analyses of variability in diagnostic accuracy, distinguishing pre-specified from exploratory	14
	**18**	Intended sample size and how it was determined	4-5, All available CTS Chagas samples
**RESULTS**			
Participants	**19**	Flow of participants, using a diagram	S2 Figure
	**20**	Baseline demographic and clinical characteristics of participants	S1 Table, S3 Table
	**21a**	Distribution of severity of disease in those with the target condition	Fig 2
	**21b**	Distribution of alternative diagnoses in those without the target condition	Not applicable

	**22**	Time interval and any clinical interventions between index test and reference standard	Not applicable<br>Banked samples
Test results	**23**	Cross tabulation of the index test results (or their distribution)<br>by the results of the reference standard	Table 1<br>Fig 3
	**24**	Estimates of diagnostic accuracy and their precision (such as 95% confidence intervals)	Fig 3, 13-14
	**25**	Any adverse events from performing the index test or the reference standard	none
**DISCUSSION**			
	**26**	Study limitations, including sources of potential bias, statistical uncertainty, and generalisability	21, 24
	**27**	Implications for practice, including the intended use and clinical role of the index test	Proof of concept,<br>Feasibility study
**OTHER INFORMATION**			
	**28**	Registration number and name of registry	Not applicable
	**29**	Where the full study protocol can be accessed	4-5, WIRB #<br>20152816
	**30**	Sources of funding and other support; role of funders	In submission form
